# Dense and Acidic Organelle-Targeted Visualization in Living Cells: Application of Viscosity-Responsive Fluorescence Utilizing Restricted Access to Minimum Energy Conical Intersection

**DOI:** 10.1021/acs.analchem.2c04133

**Published:** 2023-03-17

**Authors:** Junya Adachi, Haruka Oda, Toshiaki Fukushima, Beni Lestari, Hiroshi Kimura, Hiroka Sugai, Kentaro Shiraki, Rei Hamaguchi, Kohei Sato, Kazushi Kinbara

**Affiliations:** † School of Life Science and Technology, Tokyo Institute of Technology, 4259 Nagatsuta-cho, Midori-ku, Yokohama, Kanagawa 226-8501, Japan; ‡ Cell Biology Center, Institute of Innovative Research, Tokyo Institute of Technology, 4259 Nagatsuta-cho, Midori-ku, Yokohama, Kanagawa 226-8503, Japan; § Faculty of Pure and Applied Sciences, University of Tsukuba, 1-1-1 Tennodai, Tsukuba, Ibaraki 305-8573, Japan; ∥ Living Systems Materialogy (LiSM) Research Group, International Research Frontiers Initiative (IRFI), Tokyo Institute of Technology, 4259, Nagatsuta-cho, Midori-ku, Yokohama, Kanagawa 226-8501, Japan

## Abstract

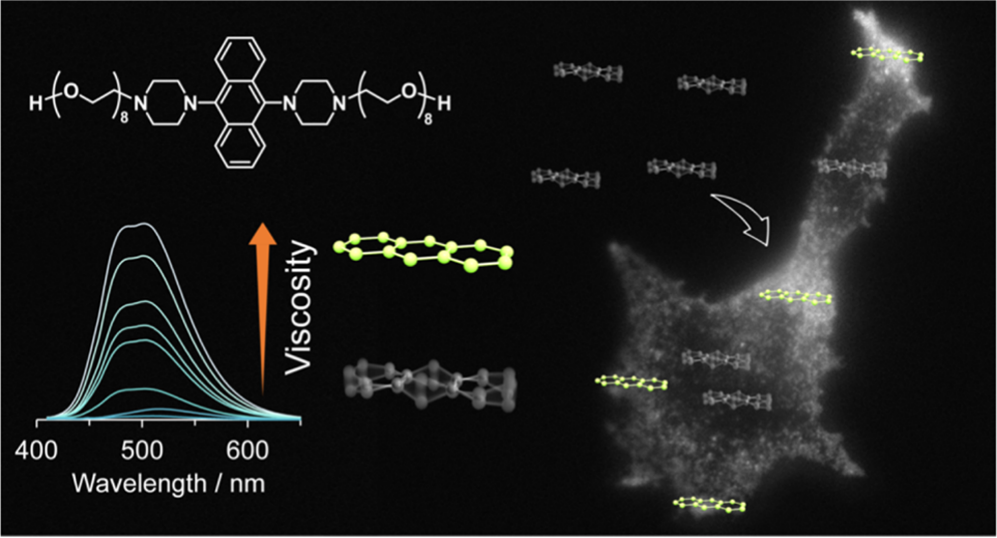

Cell-imaging methods with functional fluorescent probes are an indispensable technique to evaluate physical parameters in cellular microenvironments. In particular, molecular rotors, which take advantage of the twisted intramolecular charge transfer (TICT) process, have helped evaluate microviscosity. However, the involvement of charge-separated species in the fluorescence process potentially limits the quantitative evaluation of viscosity. Herein, we developed viscosity-responsive fluorescent probes for cell imaging that are not dependent on the TICT process. We synthesized **AnP**_**2**_**-H** and **AnP**_**2**_**-OEG**, both of which contain 9,10-di(piperazinyl)anthracene, based on 9,10-bis(*N*,*N*-dialkylamino)anthracene that adopts a nonflat geometry at minimum energy conical intersection. **AnP**_**2**_**-H** and **AnP**_**2**_**-OEG** exhibited enhanced fluorescence as the viscosity increased, with sensitivities comparable to those of conventional molecular rotors. In living cell systems, **AnP**_**2**_**-OEG** showed low cytotoxicity and, reflecting its viscosity-responsive property, allowed specific visualization of dense and acidic organelles such as lysosomes, secretory granules, and melanosomes under washout-free conditions. These results provide a new direction for developing functional fluorescent probes targeting dense organelles.

Biological events involve various types of molecules in diverse environments, but these events are generally unobservable unless a combination of spectroscopic and microscopic techniques is used.^1,2^ Cell-imaging methods with fluorescent molecular probes are indispensable for observing biological molecular behavior and have provided a better understanding of molecular cell biology. Of the various fluorescent probes (e.g., nanoparticles, polymers, and genetically encoded tags) developed to date, fluorescent small organic molecules are particularly attractive in terms of biocompatibility, ease of modification, and reproducibility.^3−5^ Considerable effort over the past several decades has led to functionalized fluorescent probes being more widely used for the selective visualization of physical parameters in cellular microenvironments.^3−7^

Microviscosity has attracted considerable attention as a physical parameter in biology because it affects several biologically important phenomena, such as diffusion condensation in the cell and protein folding.^8^ Microviscosity is most often visualized using so-called “molecular rotors”.^9−24^ Typically, molecular rotors have two energy local minima in the excited state: the locally excited (LE) state and the twisted intramolecular charge transfer (TICT) state. Since a conformational change is necessary for transition from the LE to the TICT state, higher viscosities, which restrict molecular motions, tend to suppress the transition to TICT, resulting in viscosity-responsive fluorescence.^12,25−29^ However, since the TICT state inevitably involves a charge-separated species, the fluorescence process can also be affected by other factors such as pH and the concentration of salts,^28,30,31^ which potentially limits the quantitative evaluation of viscosity in cellular environments.

To avoid such undesired sensitivity to other factors, several approaches have been adopted recently for developing viscosity-responsive fluorescent molecules that are independent of TICT. Saito and co-workers reported viscosity-sensitive flapping molecules (FLAPs) based on an excited-state planarization strategy.^32,33^ These molecules are remarkably insensitive to polarity, but the large hydrophobic aromatic moiety could make them unsuitable for use in cellular systems. Another approach is the utilization of tetraarylethene derivatives, which are known as aggregation-induced emission luminogens (AIEgens), as viscosity-responsive probes.^34,35^

In this study, we focused on viscosity-responsive fluorescence caused by restricted access to minimum energy conical intersection (MECI).^36−43^ If a large conformational change is required to access the MECI in the excited state, fluorescence of the molecules should be responsive to viscosity since high viscosity of the surrounding environment restricts the transition to the MECI. To demonstrate the application of this strategy for developing viscosity-responsive fluorescent probes that can work in cellular systems, we chose 9,10-bis(*N*,*N*-dialkylamino)anthracene, reported by Konishi and co-workers.^41,43−46^ They showed by a theoretical study that this molecule adopts a Dewar-benzene-like nonflat structure at the MECI due to a large structural change from the planar geometry at the Frank–Condon state, together with experimentally confirmed viscosity responsiveness of this molecule.^41,47^ We expected that its smaller molecular size makes it possible to design highly biocompatible molecules.

Herein, we selected acidic organelles as targets for visualizing the intracellular viscous environment, since the most viscous organelle in the cell is a lysosome,^19,34^ which is also known as the most acidic.^48^ Membrane permeability and low cytotoxicity are crucial issues when choosing molecules for the fluorescence imaging of living cells. We previously reported multiblock amphiphilic compounds consisting of aromatic hydrophobic units and hydrophilic oligo(ethylene glycol) chains that have high affinity to lipid bilayer membranes.^49^ Some of these molecules also work as transmembrane transporters.^50^ Since oligo(ethylene glycol) units are biocompatible,^51^ we designed the multiblock molecule **AnP**_**2**_**-OEG** by combining a diaminoanthracene unit with octa(ethylene glycol) (OEG) chains to allow high membrane permeability while maintaining low cytotoxicity (Figure 1). Indeed, **AnP**_**2**_**-OEG** showed high water solubility, low cytotoxicity, efficient cellular uptake, and specific visualization of dense and acidic organelles (lysosomes, regulatory secretory granules, and melanosomes) without the need to remove **AnP**_**2**_**-OEG** from culture medium. Our results provide a new design strategy for reliable viscosity-sensitive fluorescent probes independent of the TICT process.

**Figure 1:**
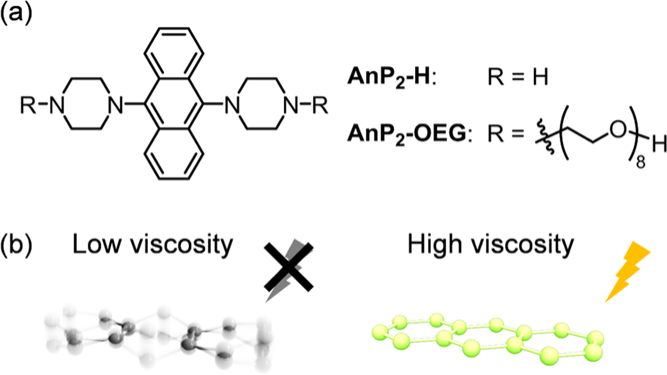
(a) Molecular structures of **AnP**_**2**_**-H** and **AnP**_**2**_**-OEG**. OEG denotes octa(ethylene glycol). (b) Schematic illustration of the anthracene unit after excitation at low and high viscosities.

## Experimental Section

### General

Column chromatography was performed using a Chromatorex NH-DM 1020 (100–200 mesh). Proton (^1^H) and carbon (^13^C) nuclear magnetic resonance (NMR) spectra were recorded on a Bruker BioSpin Avance III 400 spectrometer (^1^H: 400 MHz, ^13^C: 100 MHz). Chemical shifts are given as δ (ppm) relative to tetramethylsilane. Splitting patterns are designated as follows: s (singlet), d (doublet), t (triplet), m (multiplet), and br (broad). High-resolution mass spectrometry (HRMS) spectra were obtained using a Bruker MicrOTOF II spectrometer for electrospray ionization (ESI). Optical spectra were recorded on a Jasco V-650 spectrometer for UV–vis absorption and a Jasco FP-6500 spectrometer for fluorescence using a quartz cell with a 10 mm optical path length. Quantum yields were measured by an absolute method using a Jasco FP-8550 spectrometer equipped with an integrating sphere. An acid–base titration was performed on a Horiba model LAQUA F-72 desktop pH meter equipped with a 9618S-10D micro ToupH electrode.

### Materials

All reaction reagents and solvents were obtained from Nacalai Tesque, Fujifilm Wako, Tokyo Chemical Industry, Kanto Chemical, and Aldrich and used without further purification. Workup and purification procedures were carried out with reagent-grade solvents under air. Optical spectra were measured with spectroscopic grade solvents. Deionized water (filtered through a 0.22 μm membrane filter, *R* > 18.2 MΩ cm) was purified using a Milli-Q system from Millipore.

The reagents for cellular experiments were as follows: phosphate-buffered saline (PBS; TaKaRa), Dulbecco’s modified Eagle medium (DMEM; Nacalai Tesque), FluoroBrite DMEM (Nacalai Tesque), Opti-MEM I Reduced Serum Medium (Gibco), Trypsin-EDTA (Nacalai Tesque), MG132 (Peptide Institute, INC.), bafilomycin A1 (Merck), Lipofectamine 2000 (Invitrogen), MitoTracker Red (Thermo Fisher Scientific), Transferrin-Alexa 594 (Thermo Fisher Scientific), LumiTracker Lyso Red (LysoTracker-Red; Lumiprobe), 35 mm single-well glass-base dishes (Iwaki), and Easy iMatrix-511 for laminin coating (Nippi).

### Spectral Measurements

Stock solutions of **AnP**_**2**_**-H** and **AnP**_**2**_**-OEG** (10 mM in DMSO) were stored at −20 °C until use. Absorption and fluorescence spectra were typically obtained by adding 1.5 μL of stock solution to 3.0 mL of solvent and stirring well (final concentration of 5.0 μM). When using highly viscous solvents, the solutions were stirred at 70 °C to achieve sufficient mixing.

### Computational Methods

DFT and TD-DFT calculations were carried out using the Gaussian 16, Revision B.01 program package^52^ with the 6-31+G(d,p) basis set.^53−56^ Geometries in the ground state and the first singlet excited state were optimized by (TD-)DFT calculations with the ωB97XD^57^ functional and integral equation formalism polarizable continuum model (IEF-PCM)^58^ method for the solvation effect. The MECI was optimized at the CASSCF^59^/def2-SVP^60^ level of theory along with the def2/J auxiliary basis set,^61^ as implemented in the ORCA program package.^62^ For further details, see the Supporting Information.

### Cell Culture

HeLa, B16-F1, and AtT-20 cells were grown in DMEM supplemented with 10% fetal bovine serum (FBS), 2 mL of l-glutamine, 100 units/mL penicillin, and 0.1 mg/mL streptomycin at 37 °C and 5% CO_2_. Cytotoxicity was measured using Cell Counting Reagent SF (Nacalai Tesque).

### Plasmid Construction

We constructed plasmids for the expression of N-terminal mCherry-tagged ubiquitin (mCherry-ubiquitin) and C-terminal mCherry-tagged Phogrin and Tyrp1 (Phogrin-mCherry and Tyrp1-mCherry). Mouse Phogrin and Tyrp1 cDNAs were amplified using reverse transcription-polymerase chain reaction (RT-PCR) from mRNAs prepared from AtT-20 cells and brain tissues. These cDNAs and mCherry cDNA were subcloned into the pCMV vector.

### Plasmid Transfection

Transfections were performed using Lipofectamine 2000 according to the standard protocol. Cells were incubated for 24 h before microscopic observation.

### Preparation of Lysate

HeLa cells were lysed with lysis buffer (150 mM Tris-HCl (pH 7.4), 150 mM NaCl, 1 mM EDTA, 1 mM EGTA, and protease inhibitor cocktail (Sigma, P8340)). The lysate was homogenized with a Dounce-type homogenizer and centrifuged at 13,500 rpm for 10 min at 4 °C. The supernatant was restored. The protein concentration was determined as 21 mg/mL by the Bradford method. It was diluted by Tris buffer for fluorescence measurements. BSA was purchased from Wako Chemical. The fluorescence intensity was measured using microplate readers (Varioskan LUX, Thermo Fisher Scientific, λ_ex_ = 396 nm, λ_fl_ = 510 nm).

### Microscopic Observation

Cells were cultured in DMEM on a 35 mm glass-base dish (surface-treated with laminin for AtT-20) at 37 °C with 5% CO_2_ for 1 day. The medium was changed to FluoroBrite before microscopic observation.

Images were obtained with an inverted microscope Ti-E (Nikon) with a phase-contrast system using built-in software (NIS-elements, version 3.22, Nikon). The system comprised a PlanApo 100× VC oil immersion objective lens (NA 1.40) equipped with an EM-CCD (iXon+, gain: 5.1×, readout speed: 3 MHz, Andor) with filter sets (FF01-390/20, FF409-Di03, and FF01-525/45 for **AnP**_**2**_**-OEG**; FF01-561/14, Di02-R561, and FF01-609/54 for mCherry or Alexa549; Semrock). A xenon lamp was used as a light source.

### Staining Using the Fluorescent Probes

**AnP**_**2**_**-OEG** was dispensed as a 4 μL aliquot of a 5 mM solution in sterile water and stored at −30 °C until use. The **AnP**_**2**_**-OEG** solution was diluted with culture medium and added to cells on the glass dishes. Unless otherwise noted, the cells were then incubated at 37 °C with 5% CO_2_ for 30 or 60 min with **AnP**_**2**_**-OEG**. Commercially available probes were used in the same way with optimized concentrations. The acquired images were analyzed using ImageJ (NIH)^63^ or NIS-elements AR (ver. 5.30, Nikon).

## Results and Discussion

### Synthesis of **AnP**_**2**_**-H** and **AnP**_**2**_**-OEG**

Figure 1a shows the structure of **AnP**_**2**_**-H** and **AnP**_**2**_**-OEG** used in this work. First, we synthesized bispiperazine-substituted anthracene **AnP**_**2**_**-H** by Buchwald–Hartwig amination^64^ of 9,10-dibromoanthracene in the presence of an excess amount of piperazine. We anticipated that **AnP_2_-H** could be converted into various derivatives due to the free secondary amino groups, which can be easily functionalized without loss of fluorescence properties. **AnP**_**2**_**-H** was treated with mono-tosylated octa(ethylene glycol) in CH_3_CN in the presence of K_2_CO_3_, yielding **AnP**_**2**_**-OEG**.^65^ For details of the synthesis and characterization of **AnP**_**2**_**-H** and **AnP**_**2**_**-OEG**, see the Supporting Information.

### Photophysical Properties of **AnP**_**2**_**-OEG**

Prior to using **AnP**_**2**_**-OEG** in living cell systems, we studied its photophysical properties in aqueous solutions of different pHs to explore the effect of protonation of the amino groups on the compound’s fluorescence properties. Acid–base titration (Figure S5) of **AnP**_**2**_**-OEG** showed only one equivalence point at pH = 10.1, indicating that this molecule is weakly basic. It was reported that the second protonation of 1-methyl-4-phenylpiperazine occurs only in the concentrated acid solution (estimated as p*K*_a_ ≈ 0.7).^69^ Since the titration curve shown in Figure S5 shows good agreement with this report, we considered that the protonation occurs only at the outside (OEG-substituted) amino group in the cellular environment.

The absorption spectra of **AnP**_**2**_**-OEG** at pH = 7.4 and pH = 10.6 (Figure S6), corresponding to the protonated and deprotonated states, respectively, showed similar spectral profiles regardless of protonation/deprotonation. On the other hand, fluorescence showed a bathochromic shift with deprotonation.

Deprotonation of the amino group reportedly allows for photoinduced electron transfer (PET) and turns off the fluorescence of the adjacent chromophore in some pH-responsive fluorescent probes.^17,19,29,70,71^ However, no turnoff of fluorescence was observed for the deprotonated state of **AnP**_**2**_**-OEG**, indicating that PET does not occur in the excited state. The large Stokes shift (*ca.* 5450 cm^–1^ in the protonated state, Table S1) of **AnP**_**2**_**-OEG** compared with general molecular motors is an attractive property of this molecule for application in microscopic observation. While **AnP**_**2**_**-OEG** showed some difference in fluorescence profiles between pH = 7.4 and pH = 10.6, where protonation/deprotonation of the amino groups takes place, the fluorescence spectra were mostly unchanged within lysosomal pH from 4.5 to 5.5 (Figure S6).^48,72,73^

**Figure 2:**
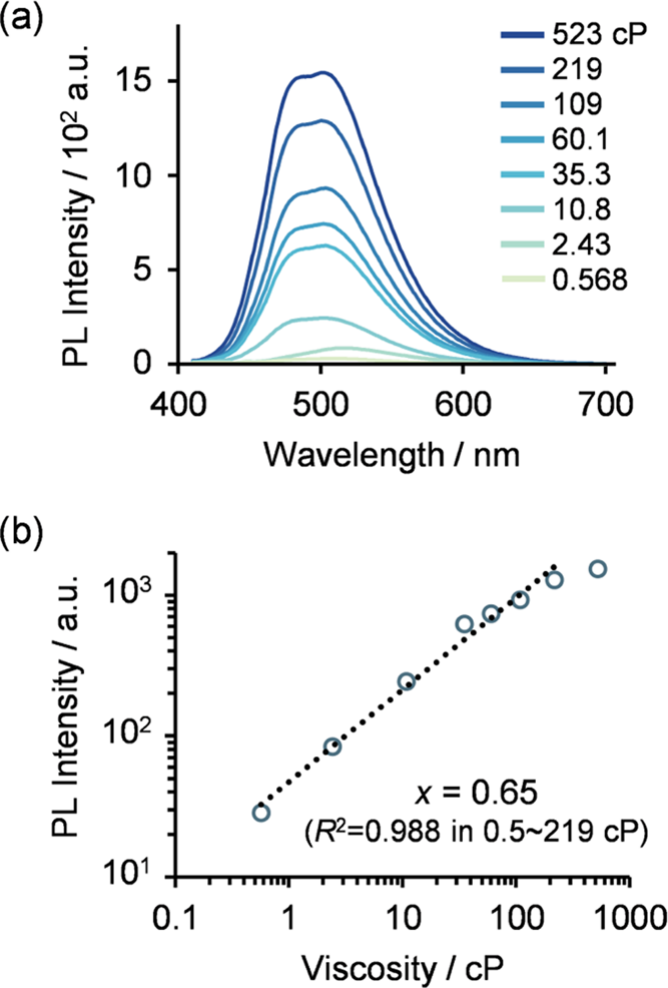
(a) Fluorescence spectra of **AnP**_**2**_**-OEG** in different solvent systems (5.0 μM at 293 K, λ_ex_ = 396 nm). (b) Relationship between the photoluminescence maximum of each spectrum and solvent viscosity. 95, 90, 85, 80, 75, and 60 w% glycerol in water, 2-propanol, and methanol, with viscosities of 523, 219, 109, 60.1, 35.3, 10.8,^66^ 2.43,^67^ and 0.568 cP,^68^ respectively, were used as the solvent. PL denotes photoluminescence.

To investigate the viscosity dependence of the fluorescence of **AnP**_**2**_**-H** and **AnP**_**2**_**-OEG**, fluorescence spectra were recorded using solvent systems with different viscosities (Figure 2a). The fluorescence intensity increased as the viscosity of the solvent increased. The relationship between fluorescence intensity and viscosity has been reported to follow the power-law relationship (the Förster–Hoffmann equation; for details, see the Supporting Information)^28,29,74,75^
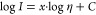
where *I* is the fluorescence intensity (or quantum yield), *x* is a dye-dependent constant used as an indicator of the sensitivity of the molecule to viscosity, η is the solvent viscosity, and *C* is a constant. The plots showed good linear relationships (Figures 2b and S8). The slope of the linear region was *x* = 0.62 (0.5–523 cP, *R*^2^ = 0.982) for **AnP**_**2**_**-H** and *x* = 0.65 (0.5–219 cP, *R*^2^ = 0.988) for **AnP**_**2**_**-OEG**, both of which are comparable to the values for TICT-based molecules (*e.g.*, *x* = 0.79 for thioflavin-T,^76^*x* = 0.56 for boron dipyrromethene (BODIPY)-based rotors,^25^ and *x* = 0.51 for julolidine-based rotors)^74^ and tetraphenylethylene-based AIEgen (*x* = 0.32).^35^ Also, the slope of both **AnP**_**2**_**-H** and **AnP**_**2**_**-OEG** is similar to those reported in Konishi’s previous study,^41^ indicating that the substitution on the side chain of diaminoanthracene does not likely affect the fluorescent properties.^73^

### Cytotoxicity of **AnP**_**2**_**-H** and **AnP**_**2**_**-OEG**

**Figure 3:**
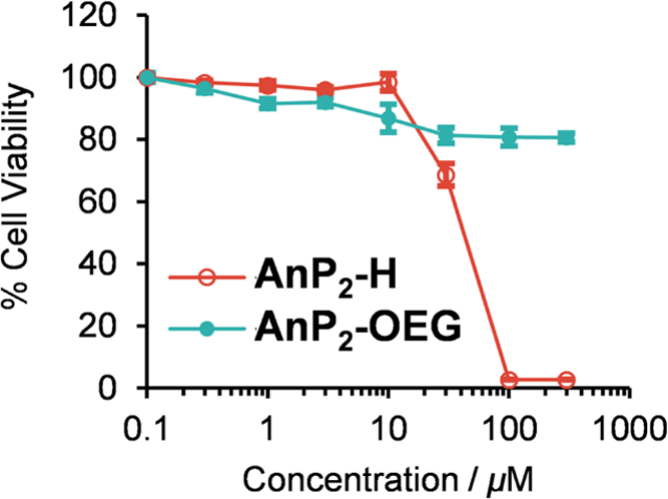
Cytotoxicity of **AnP**_**2**_**-H** and **AnP**_**2**_**-OEG**. HeLa cells were incubated in the presence of **AnP**_**2**_**-H** and **AnP**_**2**_**-OEG** for 24 h. Cell viability was evaluated by the WST-8 assay. Error bars represent standard deviations (*n* = 5).

Having confirmed that the photophysical properties of **AnP**_**2**_**-H** and **AnP**_**2**_**-OEG** are suitable for biosensing (*i.e.*, large Stokes shift and high sensitivity for viscosity), the cytotoxicity of both molecules was evaluated in cervical cancer HeLa cells using the 2-(2-methoxy-4-nitrophenyl)-3-(4-nitrophenyl)-5-(2,4-disulfophenyl)-2*H*-tetrazolium monosodium salt (WST-8) assay (Figure 3). **AnP**_**2**_**-H** was highly cytotoxic at 100 μM and above, whereas more than 80% of cells was viable after 24 h treatment with **AnP**_**2**_**-OEG** even at 300 μM. This result clearly demonstrates the importance of OEG chains for biocompatibility, and thus we chose **AnP**_**2**_**-OEG** as a fluorescent probe for exploring its application to cell imaging.

### Subcellular Localization of **AnP**_**2**_**-OEG**

Next, we examined the cellular uptake and localization of **AnP**_**2**_**-OEG** in HeLa cells. We treated HeLa cells with 10 μM **AnP**_**2**_**-OEG** and performed time-lapse imaging using phase-contrast fluorescence microscopy (Figure S12). Just after the addition of **AnP**_**2**_**-OEG**, the background intensity remained very low and a fluorescence signal was detected in the cells. The fluorescence intensity reached a steady state within 30 min, with specific spots stained in the cytoplasm. Clear fluorescence imaging required a final concentration of about 10 μM **AnP**_**2**_**-OEG**, while 1 μM was not sufficient. Washout of **AnP**_**2**_**-OEG** from the culture medium decreased the fluorescence brightness, but the fluorescent spots remained at a detectable intensity (Figure S13). This result suggested that **AnP**_**2**_**-OEG** tended to diffuse around the cell, but some was trapped in cellular compartments to some extent. A series of data collected over time demonstrate clear visualization of the cellular compartment using **AnP**_**2**_**-OEG** in washout-free conditions.

**Figure 4:**
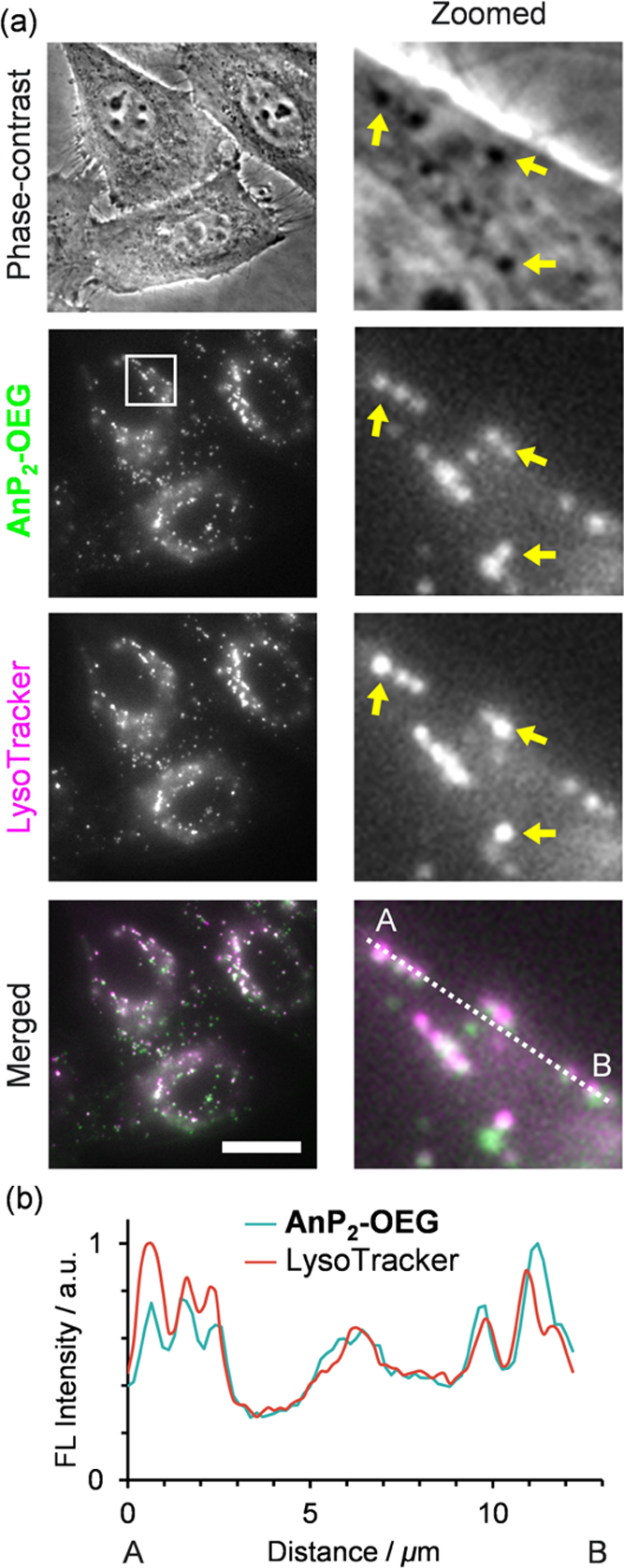
Representative microscopic images showing lysosomal staining of HeLa cells in the presence of **AnP**_**2**_**-OEG**. (a) Phase-contrast and fluorescence images of **AnP**_**2**_**-OEG** (10 μM) and LysoTracker-Red (50 nM) are shown with their merged images. Zoomed images of the boxed area are shown on the right. Arrows indicate the regions where the dark structures in the phase-contrast images coincide with fluorescence signals from both **AnP**_**2**_**-OEG** and LysoTracker. Scale bar: 20 μm. (b) Intensity profile of **AnP**_**2**_**-OEG** and LysoTracker along the dashed line.

To identify the **AnP**_**2**_**-OEG**-enriched compartments, cells were costained with typical organelle markers (*i.e.*, lysosomes, mitochondria, early endosomes, and aggresomes) (Figures 4 and S14–S16). The colocalization analysis of fluorescence signals (Figure S18) clearly showed that **AnP**_**2**_**-OEG** fluorescence was colocalized with LysoTracker (Pearson’s correlation *R* = 0.878 ± 0.029), indicating its specific visualization at lysosomes. In addition, spots with **AnP**_**2**_**-OEG** and LysoTracker fluorescence were often identified as dark structures (*i.e.*, dense structures with a high refractive index) in phase-contrast images (shown by the arrows in Figure 4 and S12). Thus, **AnP**_**2**_**-OEG** can detect lysosomes, a highly dense organelle,^77^ with low background fluorescence even in probe-containing medium. Here, we also found that the fluorescence intensity of **AnP**_**2**_**-OEG** in lysosomes reversibly changed depending on the osmolality of the medium (Figure S19). Under a hypotonic condition, the fluorescence intensity of **AnP**_**2**_**-OEG**, but not LysoTracker, became weaker. When the medium was replaced with a normal medium with physiological osmolality, **AnP**_**2**_**-OEG** fluorescence was restored to its original level. As lysosomes rapidly enlarge under hypotonic conditions and the density of internal biomolecules seems to decrease,^78^ these results indicate that fluorescence of **AnP**_**2**_**-OEG** is viscosity-responsive. In addition, the fluorescence intensity of **AnP**_**2**_**-OEG** was not significantly enhanced by the presence of proteins (Figure S20), suggesting that **AnP**_**2**_**-OEG** respond to the viscosity of the surrounding environment, enabling organelle-specific visualization.

**Figure 5:**
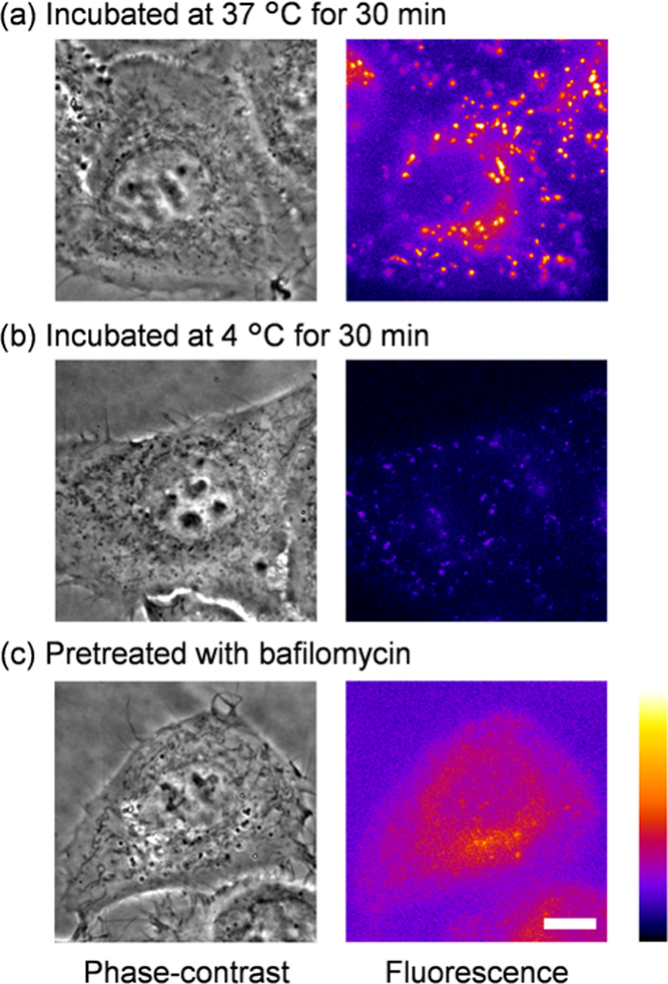
Investigation of mechanisms of cellular uptake and lysosomal localization of **AnP**_**2**_**-OEG**. Phase-contrast (left) and fluorescence (right) microscopic images of HeLa cells treated with **AnP**_**2**_**-OEG** (10 μM) at (a) 37 °C and (b) 4 °C for 30 min and (c) pretreated with bafilomycin A1 (200 nM, 37 °C, 1 h) and then treated with **AnP**_**2**_**-OEG** (10 μM, 37 °C, 30 min). Scale bar: 10 μm. Pseudo-color, lookup table (LUT): fire.

We then examined the mechanism of cellular uptake of **AnP**_**2**_**-OEG**. Small molecules generally enter living cells through two main pathways: membrane permeation and endocytosis.^79^ Endocytosis is inhibited at 4 °C.^80^ Incubation of HeLa cells with **AnP**_**2**_**-OEG** at 4 °C caused a notable decrease in the overall fluorescence signal (Figure 5a,b), indicating that its uptake occurs partly through endocytosis. However, there remained detectable punctate spots at 4 °C, indicating that **AnP**_**2**_**-OEG** can also enter cells *via* the membrane permeation mechanism. This was consistent with our molecular design, which introduced OEG chains on the nitrogen of the piperazine units.

We thus next examined the mechanism underlying lysosomal visualization due to the fluorescence signal of **AnP**_**2**_**-OEG**. Lysosomes are highly acidic compartments (pH ≈ 4.5),^48^ and bafilomycin A1 (a strong inhibitor of the lysosomal proton pump V-ATPase) decreases the lysosomal acidity.^81^ Pretreatment of cells with bafilomycin A1 decreased the fluorescence of **AnP**_**2**_**-OEG** (Figure 5c), suggesting that the acidity of lysosomes is important for **AnP**_**2**_**-OEG** accumulation. This result is consistent with the general trend that molecules having weakly basic moieties tend to be distributed in lysosomes.^7,8,82^ Based on the above results, we propose the following mechanism for the visualization of lysosomes by **AnP**_**2**_**-OEG**: (1) **AnP**_**2**_**-OEG** in the cell culture medium is taken up by cells through membrane permeation and endocytosis; (2) the molecules diffuse within the cell; (3) the acidic lysosomes trap **AnP**_**2**_**-OEG** within *ca.* 30 min; and (4) viscosity-sensitive **AnP**_**2**_**-OEG** exhibits fluorescence due to the dense environment of lysosomes, resulting in clear visualization of the lysosomes in washout-free conditions.

### Visualization of Cell-Specific Organelles by **AnP**_**2**_**-OEG**

**Figure 6:**
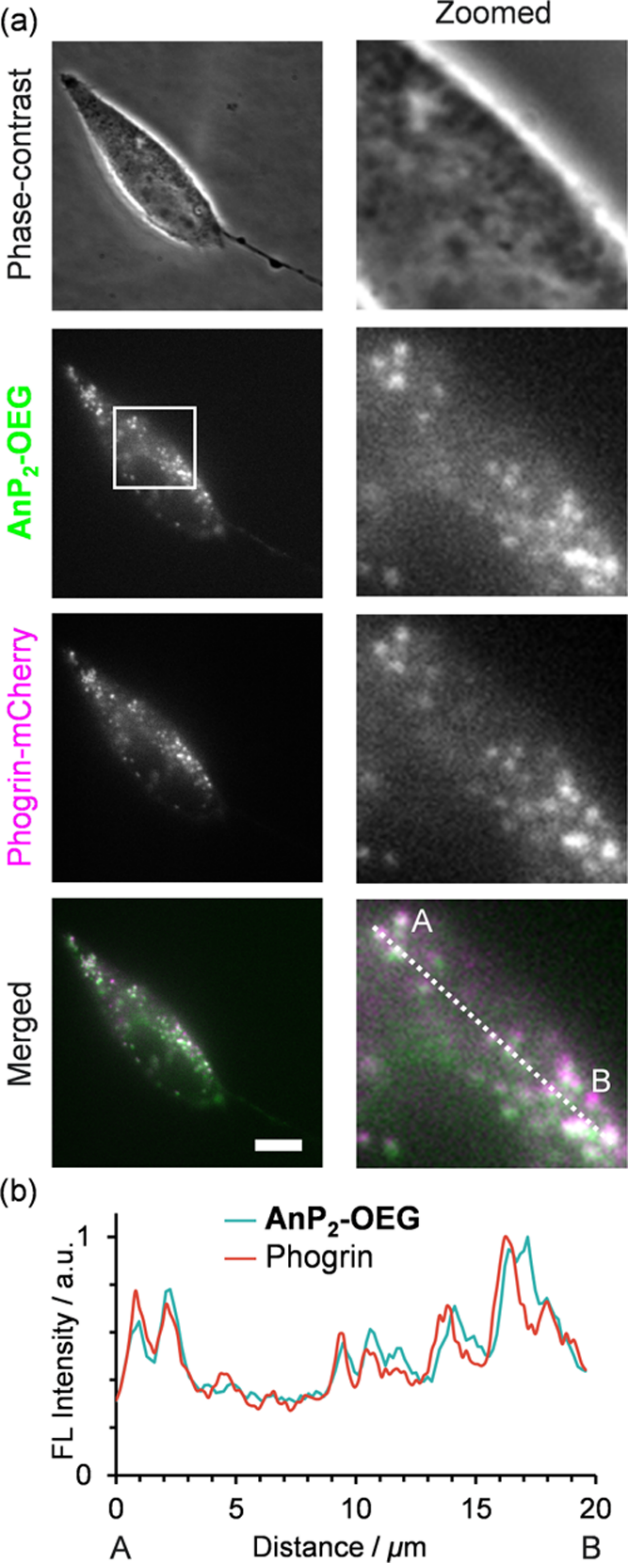
Costaining images of AtT-20 cells by **AnP**_**2**_**-OEG** and Phogrin-mCherry. (a) Phase-contrast and fluorescence images of **AnP**_**2**_**-OEG** (10 μM) and Phogrin-mCherry (expressed by transfection) are shown with merged images of **AnP**_**2**_**-OEG** and Phogrin-mCherry. Zoomed images of the boxed area are shown on the right. Scale bar: 10 μm. (b) Intensity profile of ROIs along the dashed line.

Based on the above-described mechanism, **AnP**_**2**_**-OEG** was expected to visualize not only lysosomes but also other cell-specific organelles that provide acidic and dense environments.^83^ Organelles on the regulated secretory pathway in endocrine cells gradually become more acidic and denser during maturation^48,84,85^ as secretory granules localizing near the plasma membrane. When we treated AtT-20 pituitary cells with **AnP**_**2**_**-OEG**, the fluorescence signal of **AnP**_**2**_**-OEG** was colocalized with that of mCherry-tagged Phogrin (a secretory granule marker) and the intensity profiles showed good colocalization (Figure 6). The Pearson’s correlation *R* value (*R* = 0.662 ± 0.225, Figure S18) is relatively lower than that for lysosomes in HeLa cells. Some of the **AnP**_**2**_**-OEG** signals in the perinuclear region were Phogrin-negative, suggesting that the fluorescence may also have originated from lysosomes. This result indicates that **AnP**_**2**_**-OEG** can visualize secretory granules due to their acidity and high density.

**Figure 7:**
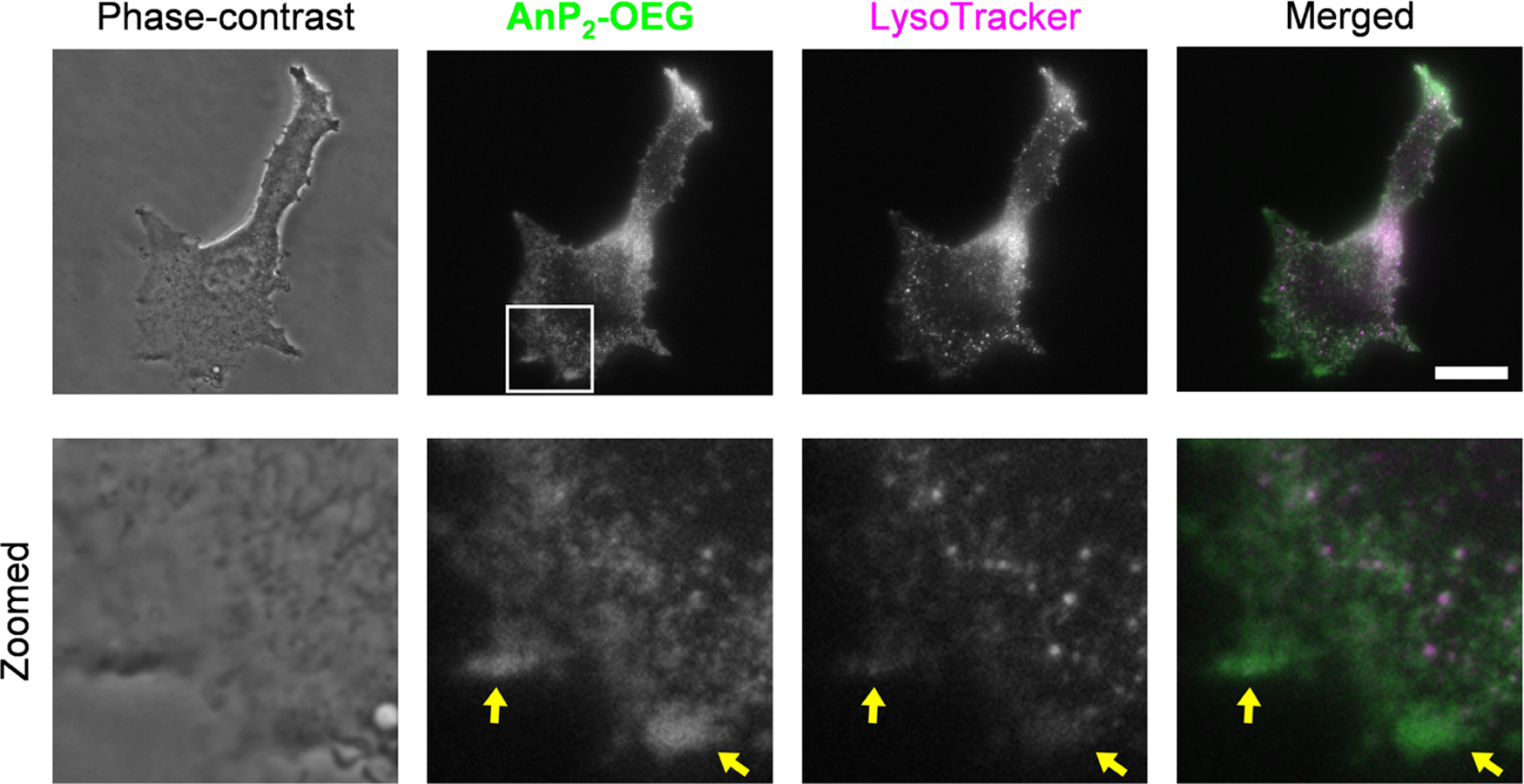
Costaining images of B16-F1 cells by **AnP**_**2**_**-OEG** and LysoTracker-Red. Phase-contrast and fluorescence images of **AnP**_**2**_**-OEG** (10 *μ*M) and LysoTracker-Red (50 nM) are shown with merged images of **AnP**_**2**_**-OEG** and LysoTracker. Zoomed images of the boxed area are shown on the bottom. Arrows indicate the **AnP**_**2**_**-OEG**-positive regions that LysoTracker rarely stained. Scale bar: 20 μm.

We next investigated melanosomes in melanin-producing cells. Melanosomes are transiently acidic^86−88^ and are among the densest organelles in a cell.^77^ Melanosomes are classified into four stages (stage I–IV) based on maturity.^89^ During maturation, their pH changes from acidic to neutral and their location changes from perinuclear to the cell peripheral region.^89,90^ When B16-F1 melanoma cells were treated with **AnP**_**2**_**-OEG**, fluorescence signals from **AnP**_**2**_**-OEG** were detected in the cytoplasm as some bright spots and along with the cell peripheral regions (Figure S17), and most of these signals were colocalized with mCherry-tagged Tyrp1, a marker mainly for stage III–IV melanosomes^89,90^ (*R* = 0.765 ± 0.073, Figure S18). Some spots, which were **AnP**_**2**_**-OEG**-positive but Tyrp1-negative, were likely lysosomes. We then compared the staining patterns of **AnP**_**2**_**-OEG** and LysoTracker. LysoTracker reportedly can stain melanosomes,^91^ but it is unclear at which stage of maturity melanosomes can be stained.^92^ We found that some **AnP**_**2**_**-OEG**-positive spots/areas in the cell peripheral region were rarely stained by LysoTracker (Figure 7, green-colored spots/areas indicated by arrows). Since LysoTracker requires an acidic environment for localization,^93^ it would not efficiently label mature melanosomes due to their low acidity.^91^ In contrast, the brighter fluorescence signal of **AnP**_**2**_**-OEG** at the cell periphery strongly suggests an advantage of **AnP**_**2**_**-OEG**: it does not require a highly acidic environment for organelle targeting and emits fluorescence in a viscosity-dependent manner.

## Conclusions

**AnP**_**2**_**-OEG** was designed as a viscosity-responsive fluorescent probe independent of the TICT process for living cells and showed viscosity-responsive fluorescence around 0.5–500 cP, efficient cellular uptake, and low cytotoxicity. Cell imaging in the presence of **AnP**_**2**_**-OEG** resulted in background-free visualization of dense and acidic organelles such as lysosomes, secretory granules, and melanosomes. Our results suggest that the viscosity-dependent fluorescence enhancement of **AnP**_**2**_**-OEG** plays an important role in this specific visualization and that weak basic moieties enhance localization at acidic organelles. To carry out the quantitative evaluation of viscosity, observation of these organelles using fluorescence lifetime imaging microscopy (FLIM), which is essentially unaffected by molecular concentration, is currently ongoing.^12,14,16,17,28,94^ We anticipate that the properties of **AnP**_**2**_**-OEG** (a small chromophore, viscosity responsiveness, efficient cellular uptake, low cytotoxicity, and independence from the TICT process) will provide a new design strategy for developing functional fluorescent probes for biological applications.

## Supporting Information Available

Details of synthesis, characterization, acid–base titration, fluorescence studies, and microscopic studies (PDF)

## Notes

An earlier version of this manuscript was submitted to the preprint server ChemRxiv: https://chemrxiv.org/engage/chemrxiv/article-details/63df6a4e45d4b84aae9e688c.
