# Supplementary material for: Dense and Acidic Organelle-Targeted Visualization in Living Cells: Application of Viscosity-Responsive Fluorescence Utilizing Restricted Access to Minimum Energy Conical Intersection
Source: Anal Chem. 2023 Mar 17;95(12):5196–204. doi: 10.1021/acs.analchem.2c04133 (PMC10061370; doi:10.1021/acs.analchem.2c04133)
Supplement: Supplementary file 1 — ac2c04133_si_001.pdf [file ac2c04133_si_001.pdf]

Supporting Information

Dense and Acidic Organelle-Targeted Visualization in Living Cells: Application of Viscosity-Responsive Fluorescence Utilizing Restricted Access to Minimum Energy Conical Intersection

Junya Adachi,[a] Haruka Oda,[b] Toshiaki Fukushima,*[a, b] Beni Lestari,[a] Hiroshi Kimura,[a, b] Hiroka Sugai,[c],† Kentaro Shiraki,[c] Rei Hamaguchi,[a] Kohei Sato,[a] Kazushi Kinbara*[a, d]

[a] School of Life Science and Technology, Tokyo Institute of Technology, 4259 Nagatsuta-cho, Midori-ku, Yokohama, Kanagawa 226-8501, Japan
[b] Cell Biology Center, Institute of Innovative Research, Tokyo Institute of Technology 4259 Nagatsuta-cho, Midori-ku, Yokohama, Kanagawa 226-8503, Japan
[c] Faculty of Pure and Applied Sciences, University of Tsukuba, 1-1-1 Tennodai, Tsukuba, Ibaraki 305-8573, Japan
[d] Living Systems Materialogy (LiSM) Research Group, International Research Frontiers Initiative (IRFI), Tokyo Institute of Technology, 4259, Nagatsuta-cho, Midori-ku, Yokohama 226-8501, Japan

*To whom correspondence should be addressed.
tofu@bio.titech.ac.jp (T.F.); kkinbara@bio.titech.ac.jp (K.K.)

Table of Contents

1. Synthesis S2
2. NMR spectra S4
3. High-resolution MS spectrometry S6
4. Acid-base titration of **AnP$_2$-OEG** S7
5. Photophysical properties of **AnP$_2$-OEG** and **AnP$_2$-H** S7
   5.1 pH dependence of absorption and fluorescence spectra S7
   5.2 Viscosity-dependent fluorescence S8
   5.3 Photophysical data of **AnP$_2$-OEG** S10
6. Theoretical study S11
   6.1 (TD-)DFT calculation S11
   6.2 MECI geometry of **AnP$_2$-Me** S12
7. Cellular experiments S15
   7.1 Time lapse imaging of HeLa cells after addition of **AnP$_2$-OEG** S15
   7.2 Effect of washout of **AnP$_2$-OEG** from the culture medium S16
   7.3 Different staining images of HeLa cells by **AnP$_2$-OEG** and MitoTracker S17
   7.4 Different staining images of HeLa cells by **AnP$_2$-OEG** and Transferrin-Alexa594 S18
   7.5 Different staining images of HeLa cells with aggresomes by **AnP$_2$-OEG** and mCherry-ubiquitin S19
   7.6 Co-staining images of B16-F1 melanoma cells by **AnP$_2$-OEG** and Tyrp1-mCherry S20
   7.7 Co-localization analysis of **AnP$_2$-OEG** with organelle markers S21
   7.8 Effects of osmolality on fluorescence intensity of **AnP$_2$-OEG** and LysoTracker S22
   7.9 Effects of proteins on fluorescence intensity of **AnP$_2$-OEG** S23
8. References S24

1. Synthesis

9,10-Di(piperazin-1-yl)anthracene (AnP$_2$-H)

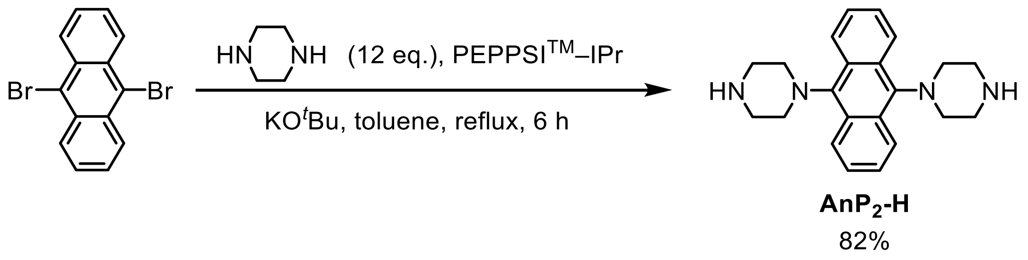

In flame-dried 30 mL 2-neck flask equipped with a magnetic stirring bar and a reflux condenser were added 9,10-dibromoanthracene (334 mg, 0.994 mmol), piperadine (anhydrous, 1.057g, 12.27 mmol), KO^{t}Bu (344 mg, 3.07 mmol) and PEPPSI-IPr (15.8 mg 0.0232 mmol), and the resulting mixture was dried under reduced pressure for 10 min. Then, to the mixture was added dry toluene (6 mL) under Ar. The resulting suspension was refluxed at 120 °C (oil bath) for 6 h. After the reaction, the reaction mixture was cooled to room temperature and quenched by water (10 mL). The organic layer was separated and the aqueous layer was extracted with toluene (15 mL ×6). The combined organic layer was washed with brine, dried over Na_2SO_4, filtrated and concentrated under reduced pressure. The residue was purified by column chromatography (NH silica, 1% ^{i}PrOH in CH_2Cl_2 as eluent) to obtain **AnP$_2$-H** (yellow solid, 283 mg, 82%); ^{1}H NMR (400 MHz, $CDCl_3$): δ 3.20 (t, J = 4.5 Hz, 8H), 3.49 (t, J = 4.5 Hz, 8H), 7.46 (AA'BB', 4H), 8.57 (AA'BB', 4H); ^{13}C NMR ($CDCl_3$, 100 MHz) δ 47.5, 52.6, 124.6, 125.3, 131.2, 142.6; HRMS–ESI (*m*/*z*): $[M+H]^+$ calcd for $C_{22}H_{26}N_4$: 347.2230, found: 347.2241.

23,23'-(Anthracene-9,10-diylbis(piperazine-4,1-diyl))bis(3,6,9,12,15,18,21-heptaoxatricosan-1-ol) (AnP$_2$-OEG)

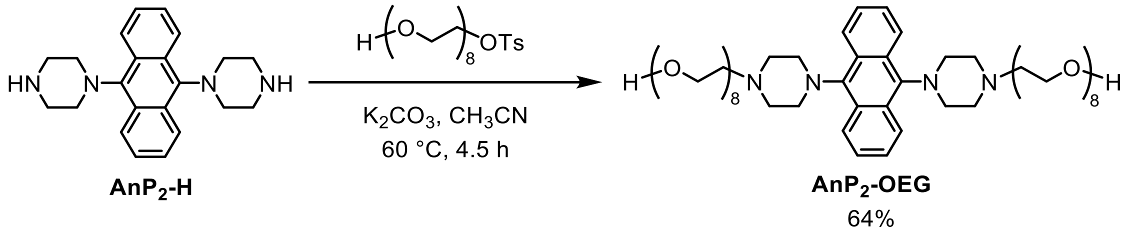

In flame-dried 50 mL 2-neck flask equipped with a magnetic stirring bar were placed tetraethylene glycol mono(*p*-toluenesulphonate) ester[S1] (609 mg, 1.16 mmol), **AnP$_2$-H** (136 mg, 0.393 mmol), K_2CO_3 (353 mg, 2.56 mmol) and dry CH_3CN (5 mL). The resulting suspension was stirred at 60 °C (oil bath) for 4.5 h. To the reaction mixture, was added ethylenediamine (200 μL)

at 50 °C to decompose the unreacted tosylate. Then, the solvent was evaporated under reduced pressure, and to the residue was added water (20 mL) followed by extraction with CH_2Cl_2 (10 mL ×5). The combined organic layer was washed with brine, dried over Na_2SO_4, filtrated and concentrated under reduced pressure. The residue was purified by column chromatography (NH silica, hexane/CH_2Cl_2/MeOH = 60/40/1 to 40/60/3 as eluent) to obtain **AnP$_2$-OEG** (yellow oil, 263 mg, 64%); ^{1}H NMR (400 MHz, $CDCl_3$): δ 2.80 (t, *J* = 5.8 Hz, 4H), 2.84 (t, *J* = 4.2 Hz, 8H), 3.54 (t, *J* = 4.2 Hz, 8H), 3.58–3.73 (m, 56H), 3.75 (t, *J* = 5.8 Hz, 4H), 7.44 (AA'BB', 4H), 8.55 (AA'BB', 4H); ^{13}C NMR ($CDCl_3$, 100 MHz) *δ* 51.1, 55.1, 58.2, 61.5, 68.9, 70.1, 70.26, 70.32, 70.35, 70.36, 70.38, 70.40, 70.44, 70.46, 72.6, 124.6, 125.2, 131.1, 142.3; HRMS–ESI (*m*/*z*): $[M+Na]^{2+}$ calcd for $C_{54}H_{90}N_4O_{16}$: 548.3068, found: 548.3051.

2. NMR spectra

(a)

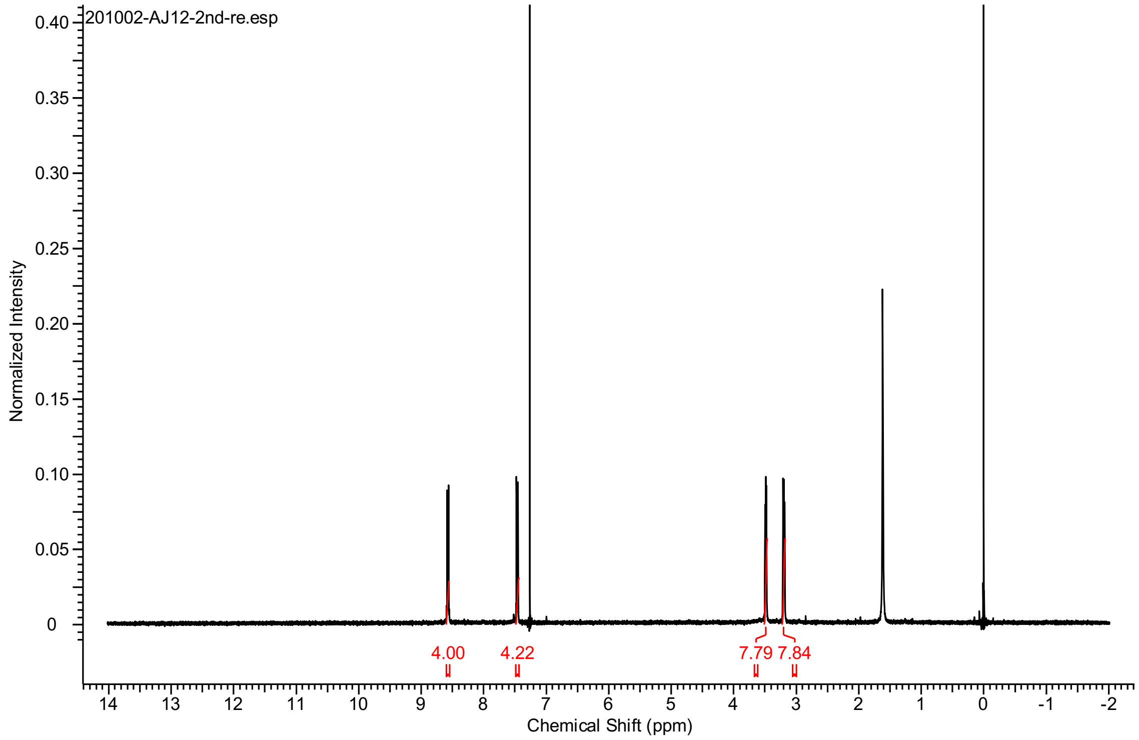

(b)

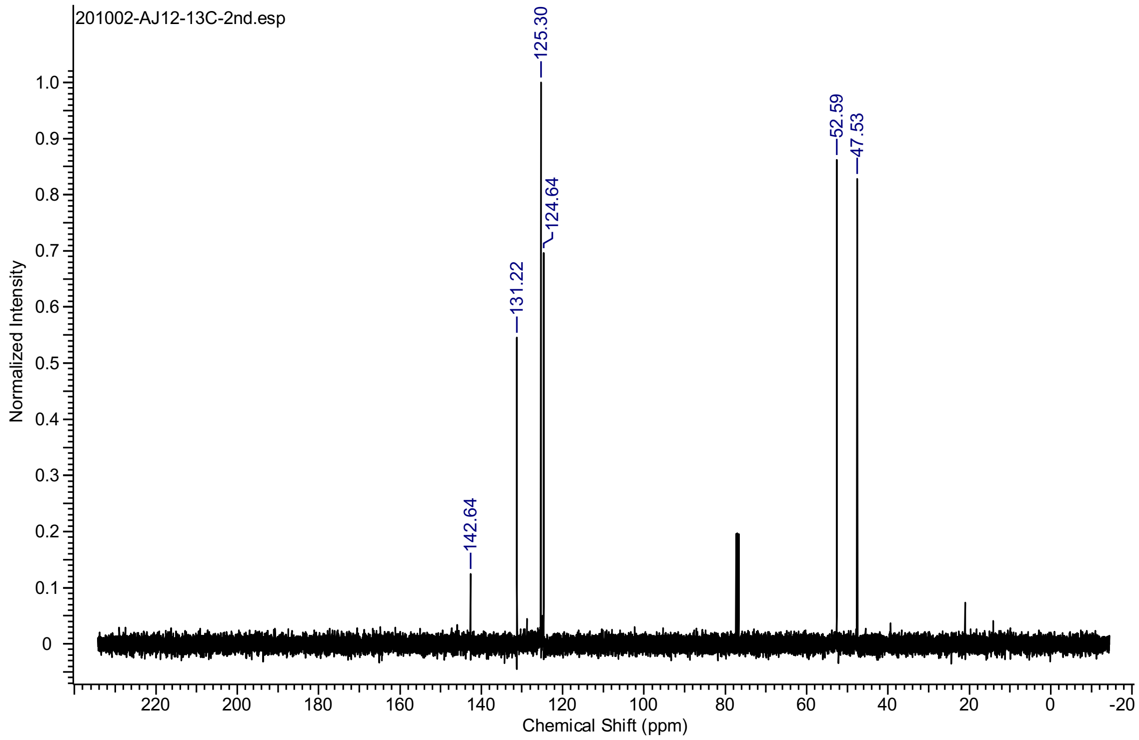

Figure S1. (a) ^{1}H and (b) ^{13}C NMR spectra of **AnP$_2$-H** in $CDCl_3$.

(a)

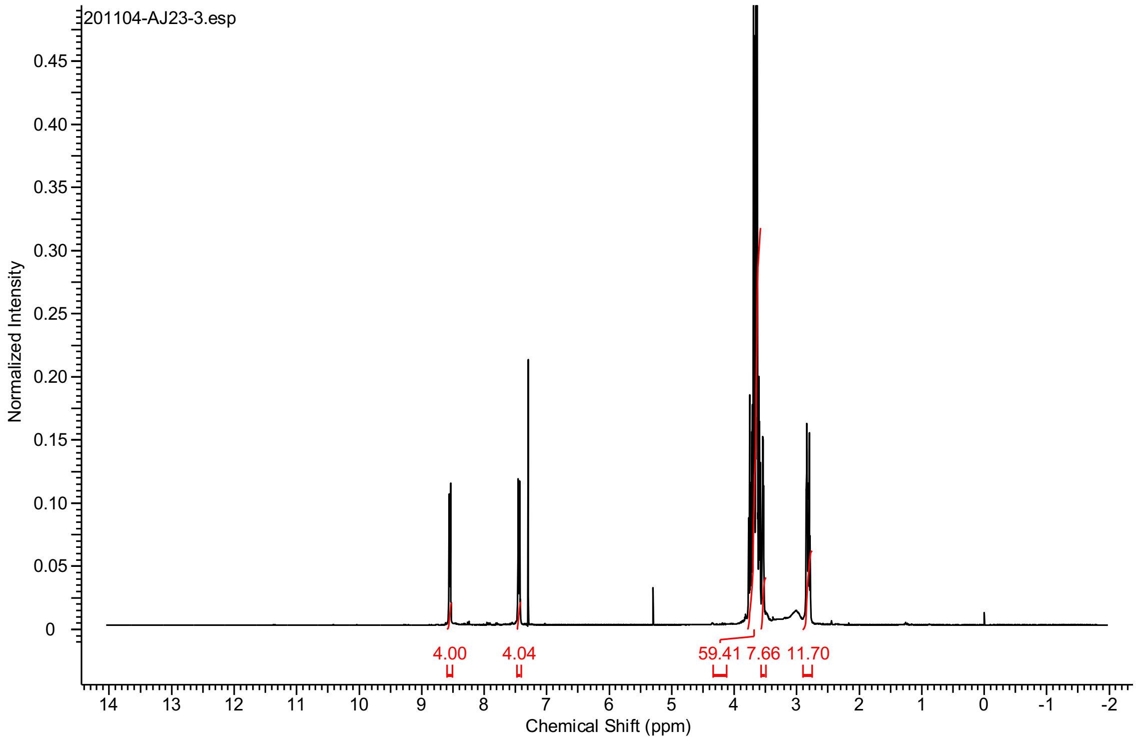

(b)

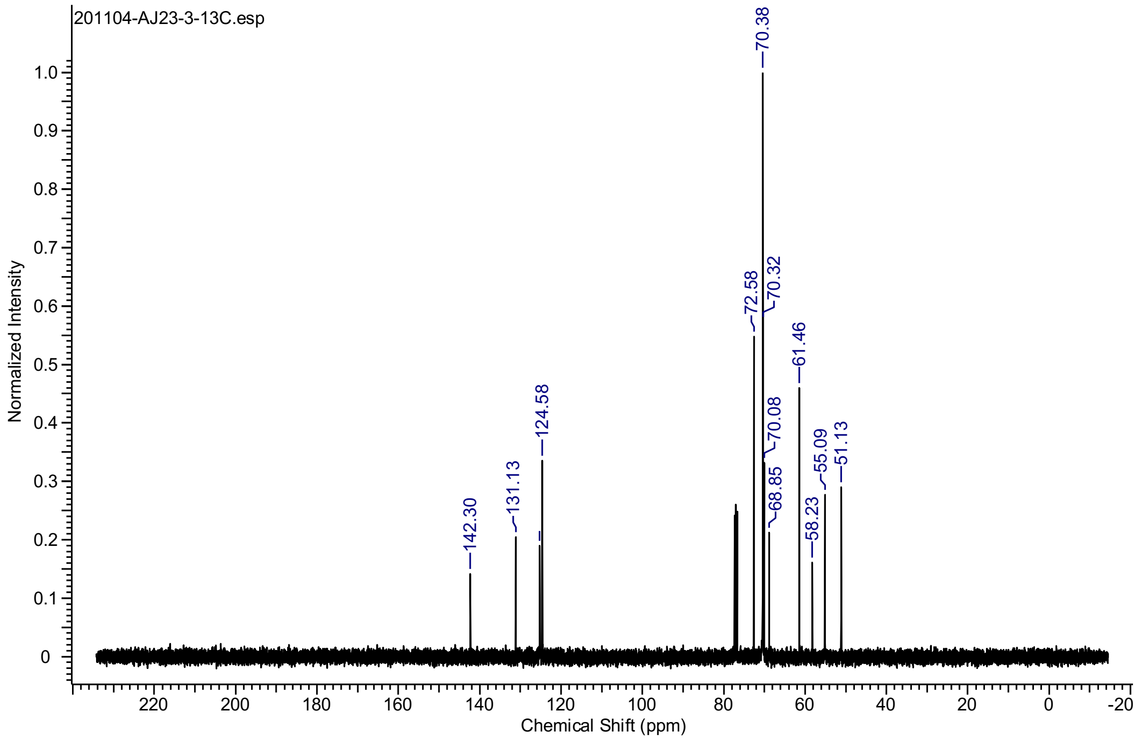

Figure S2. (a) ^{1}H and (b) ^{13}C NMR spectra of **AnP$_2$-OEG** in $CDCl_3$.

3. High-resolution MS spectrometry

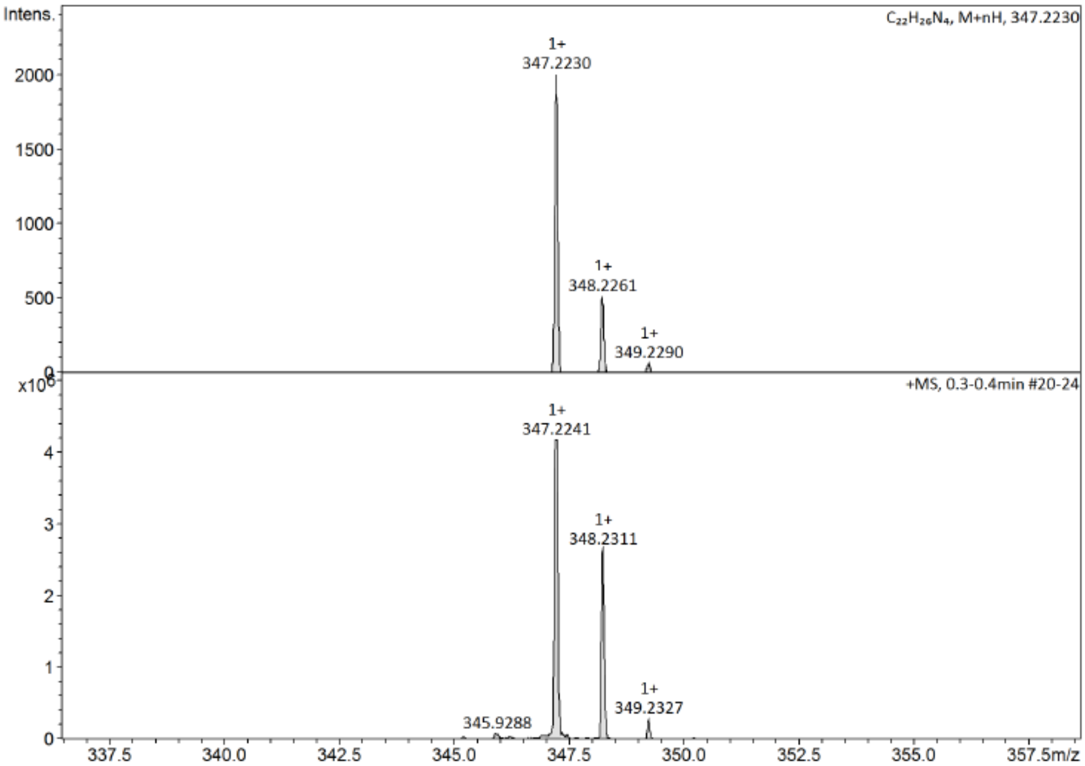

Figure S3. HRMS profile of **AnP$_2$-H**.

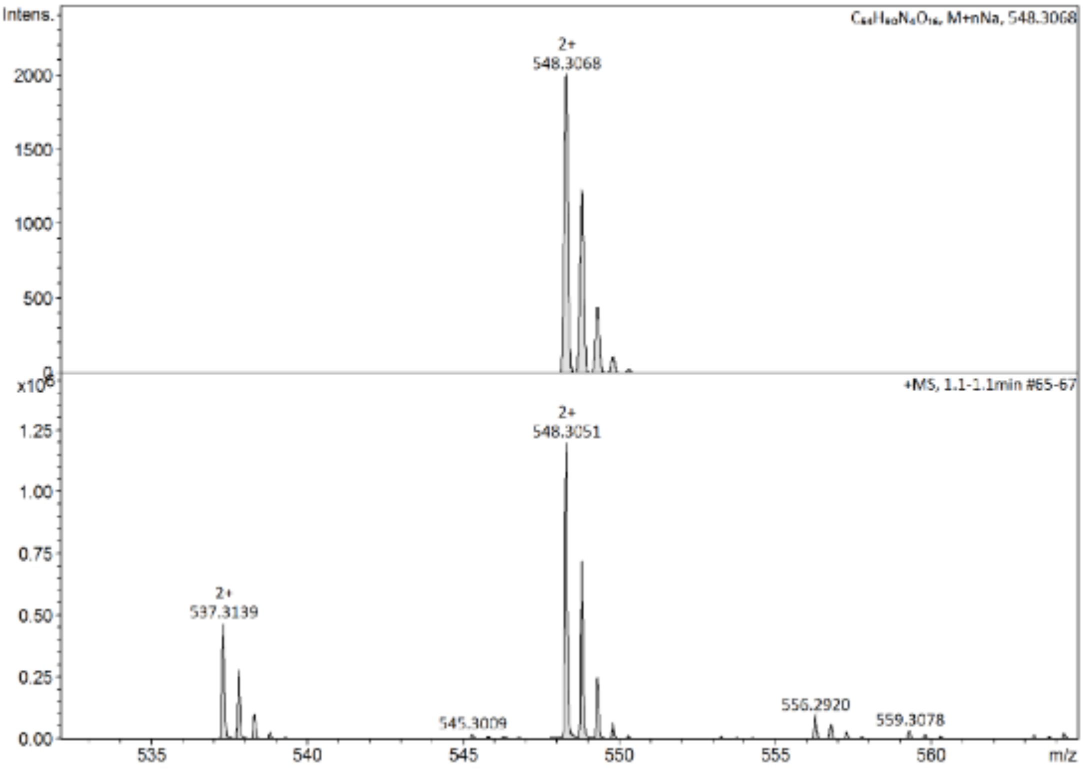

Figure S4. HRMS profile of **AnP$_2$-OEG**.

4. Acid-base titration of AnP2-OEG

AnP2-OEG (20.1 mg) was dissolved in a mixture of dimethyl sulfoxide (DMSO) (15 μL) and milli-Q water (950 μL). The resulting solution was beforehand acidified by HCl aq. (10 μL), and then titrated by an aqueous solution of NaOH (1.0 M). The pH value was monitored by a pH meter.

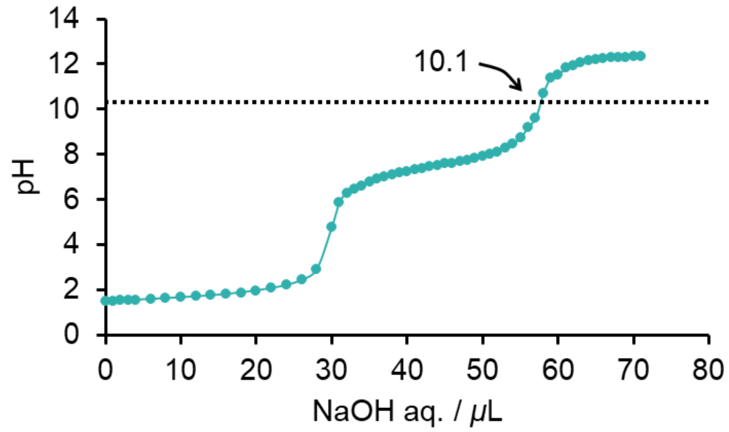

Figure S5. Titration curve of **AnP2-OEG** at room temperature. The first equivalence point at pH ≈ 4 corresponds to the neutralization of excessively added HCl.

5. Photophysical properties of AnP2-OEG and AnP2-H

5.1 pH Dependence of absorption and fluorescence spectra

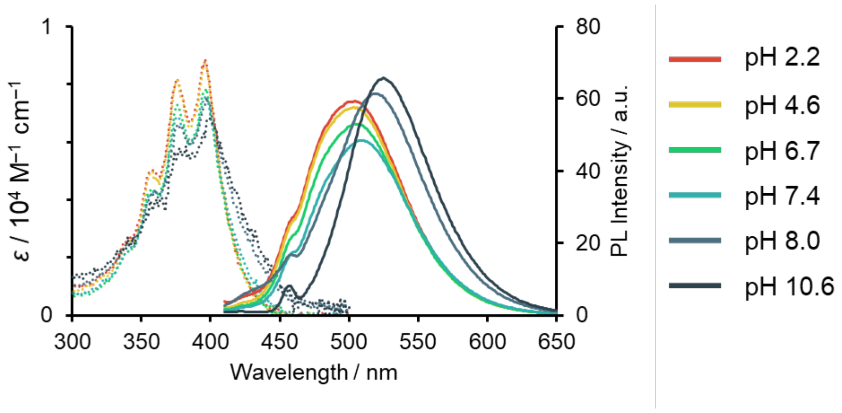

Figure S6. Absorption (dashed lines) and fluorescence (solid lines) spectra of **AnP2-OEG** at various pH values (5.0 μM at 293 K, λ_{ex} = 396 nm). The solutions were buffered to pH = 2.2, 4.6, 6.7, 7,4 8.0, and 10.6 with 100 mM of phosphate, acetate, phosphate, phosphate, Tris (tris(hydroxymethyl)aminomethane), and CAPS (*N*-cyclohexyl-3-aminopropanesulfonic acid), respectively.

5.2 Viscosity-dependent fluorescence

As described in the main text, the quantum yield Φ and solvent viscosity η follow a power-law relationship that is known as the Förster-Hoffmann equation,[S2,S3]

$$\phi = C \cdot \eta^{x} \Leftrightarrow \log \phi = x \cdot \log \eta + C' \quad (1)$$

where C (C') is a solvent dependent constant and x is a dye dependent constant which is used as an indicator of the sensitivity of the molecule to viscosity.

Using the rate constants for each decay process, fluorescence quantum yield Φ (or fluorescence intensity I) is also described as

$$I = A \cdot \phi = A \cdot \frac{k_{\mathrm{r}}}{k_{\mathrm{r}} + k_{\mathrm{nr}}} \quad (2)$$

where A is proportionality constant, k_{r} is radiative decay rate constant, and k_{nr} is non-radiative decay rate constant. The viscosity dependence has been discussed mainly associated with the twisted intramolecular charge transfer (TICT) process. The viscosity-dependent fluorescence property originates from the twisting motion of molecule occurring in transition from the LE state to the TICT state, which is affected by the viscosity of the surrounding environment. Namely, the fluorescence is exhibited from the LE state, while the transition to the TICT state causes the non-radiative deactivation depending on the viscosity (Figure S7). Here, for simplicity, equation (2) assumes the TICT state to be non-fluorescent.[S3] However, it can also be applied to dual-emissive molecules. In this case, the ratio of the intensities of the two fluorescent components is used instead of Φ.

Meanwhile, MECI is the lowest crossing point of S_0 and S_1 potential energy surface. The energy level of MECI dominates non-radiative decay rate of the excited molecules. Here, 9,10-bis(*N*,*N*-dialkylamino)anthracene undergoes a large conformational change from the fluorescent state to accessing MECI,[S4] where the deactivation through MECI is suppressed as the viscosity around the molecule increases.

Based on the above discussion, the decay processes of TICT and MECI systems were schematically shown in Figure S7. In both systems, the first local minimum on the S_1 potential surface is involved in the first relaxation (k_{r} and k_{nr}). In the case of TICT system (Figure S8a), the transition to the TICT state, allowing for non-radiative decay, requires conformational changes, thereby resulting in viscosity-dependent non-radiative decay, $k_{\mathrm{nr}}(\eta)$. Similarly, in the case of MECI system, conformational changes allow non-radiative decay through MECI, thereby also resulting in viscosity-dependent non-radiative decay, $k_{\mathrm{nr}}(\eta)$. Therefore, the viscosity dependence of the fluorescence intensity of the MECI system was also expected to satisfy equation (2).

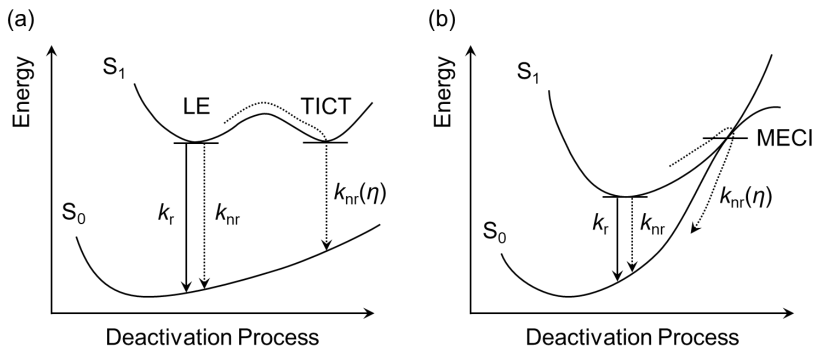

Figure S7. Schematic illustration of the decay process from the photo-excited S_1 state of (a) TICT and (b) MECI systems.

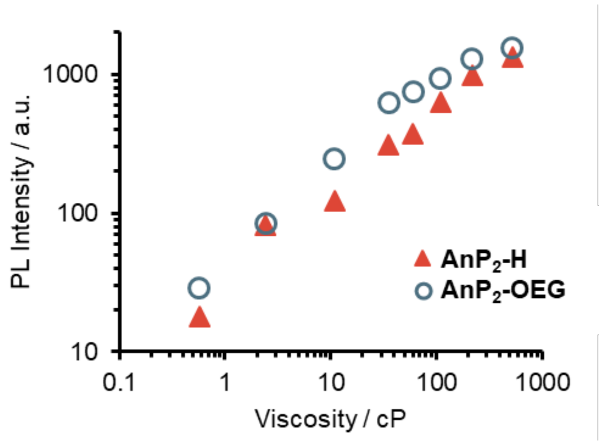

Figure S8. Förster-Hoffmann plots of **AnP₂-H** and **AnP₂-OEG**. 95 w%, 90 w%, 85 w%, 80 w%, 75 w%, 60 w% glycerol in water, 2-propanol, and methanol at 523, 219, 109, 60.1, 35.3, 10.8,[S5] 2.43,[S6] and 0.568 cP,[S7] respectively, were used as solvents (5.0 μM at 293 K, λ_{ex} = 396 nm).

5.3 Photophysical data of AnP_2-OEG

Table S1. Wavelengths at absorption (λ_{abs}) and fluorescence (λ_{fl}) maxima, Stokes shifts ($\Delta\upsilon_{St}$), and fluorescence quantum yields (Φ) for **AnP_2-OEG**

Solvent	λ_{abs} / nm	λ_{fl}[a] / nm	$\Delta\upsilon_{St}$[b] / cm^{-1}	Φ[c]
pH 7.1 buffer[d]	376, 396	505	5450	–
pH 10.6 buffer[e]	378, 399	530	6200	–
Methanol	375, 396	509	5610	0.02
90% glycerol in water	377, 398	501	5290	0.43

[a]Excitation wavelength: 396 nm.

[b]Calculated from wavelengths of the maxima of the absorption bands and that of the fluorescence.

[c]Evaluated at room temperature.

[d]20 mM HEPES and 50 mM NaCl solution.

[e]20 mM CAPS and 50 mM NaCl solution.

6. Theoretical study

6.1 (TD-)DFT calculation

The theoretical calculation has been carried out to study the reason of different emission wavelength of **AnP₂** at different pH. The molecular geometries of model compounds, **AnP₂-Me** (deprotonate state) and **AnP₂-Me-2H** (protonate state), in the ground state and the first singlet excited state were optimized at (TD-)ωB97XD/6-31+G(d,p) level with IEF-PCM in water, and the resulting geometries were used for the calculation. Jablonski diagrams of **AnP₂-Me** and **AnP₂-Me-2H**, constructed based on the result of (TD-)DFT calculation were described in Figure S9. The theoretical calculations revealed that protonation destabilizes the local minima of the S_1 state, thereby explaining the spectral blue shift of the emission of **AnP₂** at low pH of the solution. In addition, TD-DFT calculation showed that the major electronic configuration of S_1 state is formed by HOMO-to-LUMO transition, which is attributed to local π-π^* transition of the anthracene moiety. The non-bonding orbital of N atoms is at a lower energy level than the HOMO (π) orbital (Figure S10), suggesting that PET cannot occur in **AnP₂**.

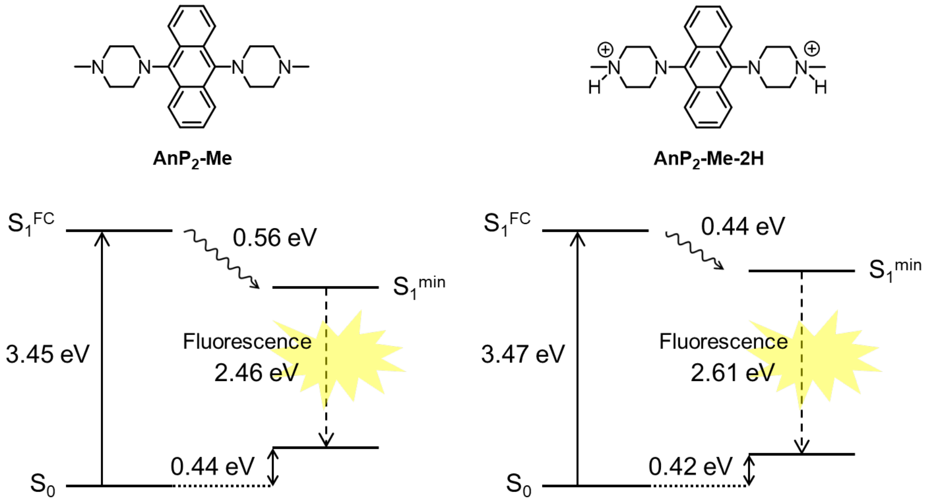

Figure S9. Jablonski diagram of **AnP₂-Me** and **AnP₂-Me-2H** estimated by (TD-)DFT calculation at ωB97XD/6-31+G(d,p) level with IEF-PCM in water.

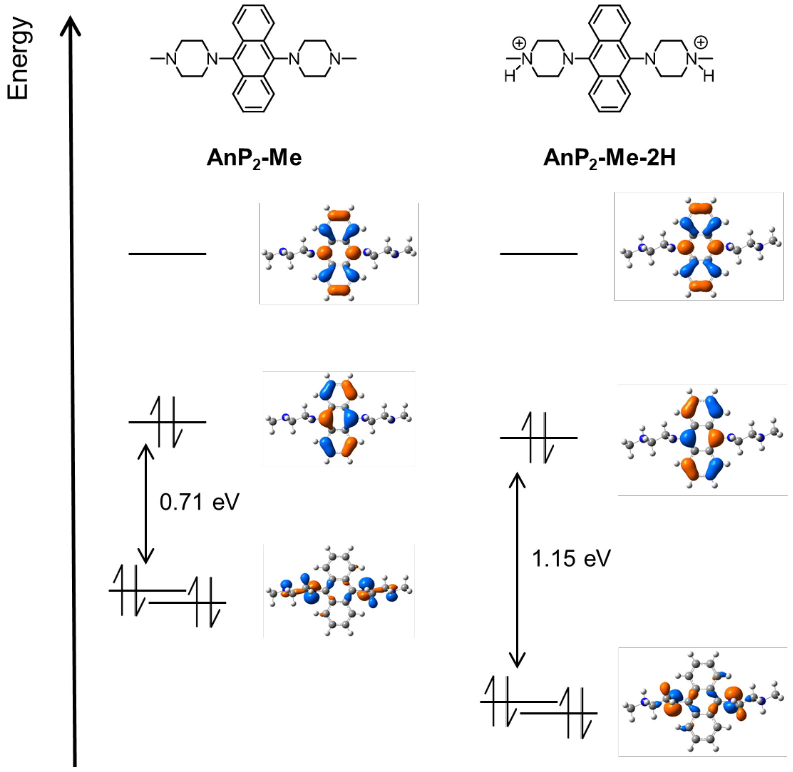

Figure S10. Selected molecular orbitals and their energy levels of **AnP$_2$-Me** and **AnP$_2$-Me-2H** estimated by (TD-)DFT calculation at ωB97XD/6-31+G(d,p) level with IEF-PCM in water based on the optimized structure.

6.2 MECI geometry of AnP$_2$-Me

The search of MECI geometry of **AnP$_2$-Me** was performed at the state-averaged complete active space self-consistent-field theory (CASSCF) level using def2-SVP basis set along with the def2/J auxiliary basis set starting from optimized S_1 geometry (BHHLYP/6-31+G(d,p)). In the obtained MECI geometry, the anthracene unit adopted a Dewar-benzene-like non-flat structure, similarly to the previous studies.[S8,S9]

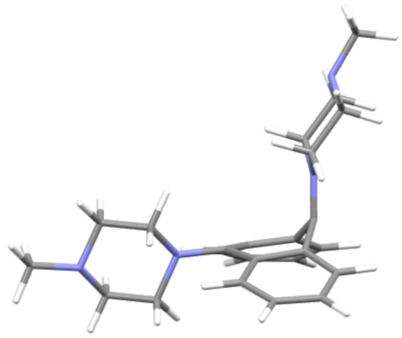

Figure S11. Searched MECI geometry of **AnP$_2$-Me** (CASSCF/def2-SVP, def2/J).

Table S2. MECI structure of **AnP$_2$-Me**

Atomic symbol	x	y	z
C	-0.09062	-0.29200	1.22335
C	1.14643	-0.02689	0.63514
C	1.76994	1.23874	0.79609
C	0.98349	2.26411	1.43444
C	-0.21082	1.96442	2.08258
C	3.17050	1.51933	0.54234
C	1.65045	3.57844	1.30802
C	2.99799	3.57635	1.92894
C	3.81814	2.59851	1.26806
C	5.21651	2.70547	1.47040
H	5.88813	2.09741	0.86057
C	4.88195	4.37876	3.21467
C	3.50475	4.38950	2.93670
H	-0.53595	-1.28353	1.09886
H	1.64791	-0.81553	0.07311
H	-0.74542	2.75482	2.61796
H	5.29403	5.03800	3.98231
H	2.83896	5.08452	3.45785
C	5.72695	3.57223	2.43869
H	6.80904	3.61973	2.59436
C	-0.76151	0.67741	1.98191
H	-1.72114	0.45006	2.45201
C	4.65434	-0.47808	0.64780
C	3.69470	0.17016	-1.48350
C	5.90683	-0.97366	-0.07322
H	3.97198	-1.34227	0.80422
H	4.92407	-0.08848	1.64018
C	4.96896	-0.30443	-2.18493
H	2.95448	-0.65802	-1.53198
H	3.26995	1.02889	-2.02589
H	6.32442	-1.83746	0.47201
H	6.68161	-0.16917	-0.05648
H	4.71467	-0.67372	-3.19345

H	5.65866	0.56422	-2.31220
C	2.35626	5.36938	-0.24704
C	0.39069	4.02487	-0.77304
C	1.61373	6.60788	-0.74542
H	2.96116	4.94258	-1.07022
H	3.03848	5.62768	0.57125
C	-0.31416	5.28748	-1.26567
H	0.87617	3.51203	-1.62630
H	-0.32847	3.32704	-0.32770
H	2.35041	7.30982	-1.16969
H	1.13718	7.12060	0.12456
H	-1.00704	5.00748	-2.07621
H	-0.93430	5.70371	-0.43562
N	1.41682	4.34942	0.21594
N	0.64035	6.26133	-1.76724
N	4.00025	0.57750	-0.12213
N	5.59670	-1.38285	-1.43552
C	6.72865	-1.96703	-2.12163
H	7.11527	-2.82778	-1.55148
H	6.42361	-2.33076	-3.11666
H	7.57414	-1.25348	-2.26832
C	0.01084	7.41844	-2.37089
H	0.77694	8.08439	-2.79960
H	-0.65689	7.10208	-3.18834
H	-0.59447	8.01705	-1.65100

7. Cellular experiments

7.1 Time-lapse imaging of cells treated with AnP$_2$-OEG

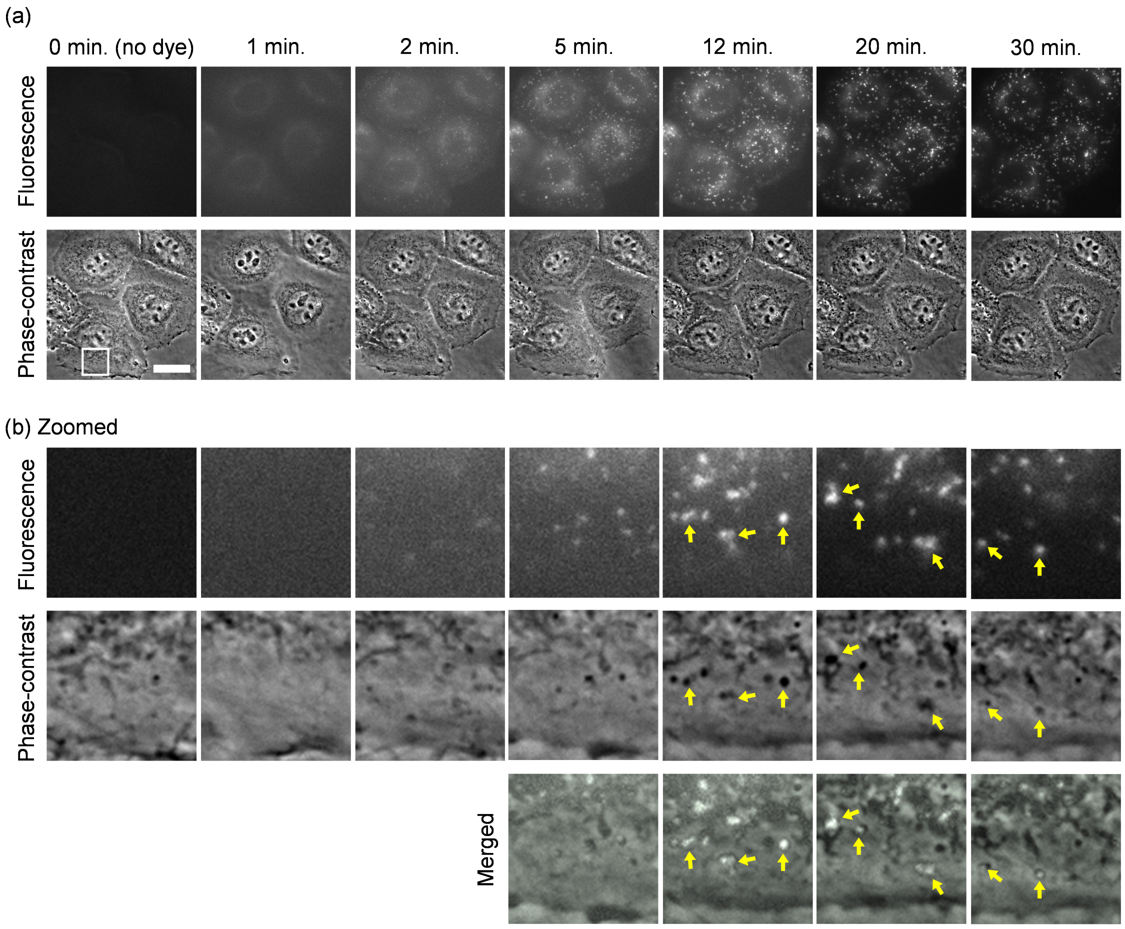

Figure S12. (a) HeLa cells were treated with **AnP$_2$-OEG** (10 μM) and subjected to time-lapse imaging analysis. Fluorescence and phase-contrast images were acquired. (b) Zoomed images of the boxed area in (a). Arrows show where the dark areas in the phase-contrast images coincide with fluorescent signals from both **AnP$_2$-OEG** and LysoTracker. Scale bar: 20 μm.

7.2 Effects of washout of AnP$_2$-OEG from the culture medium

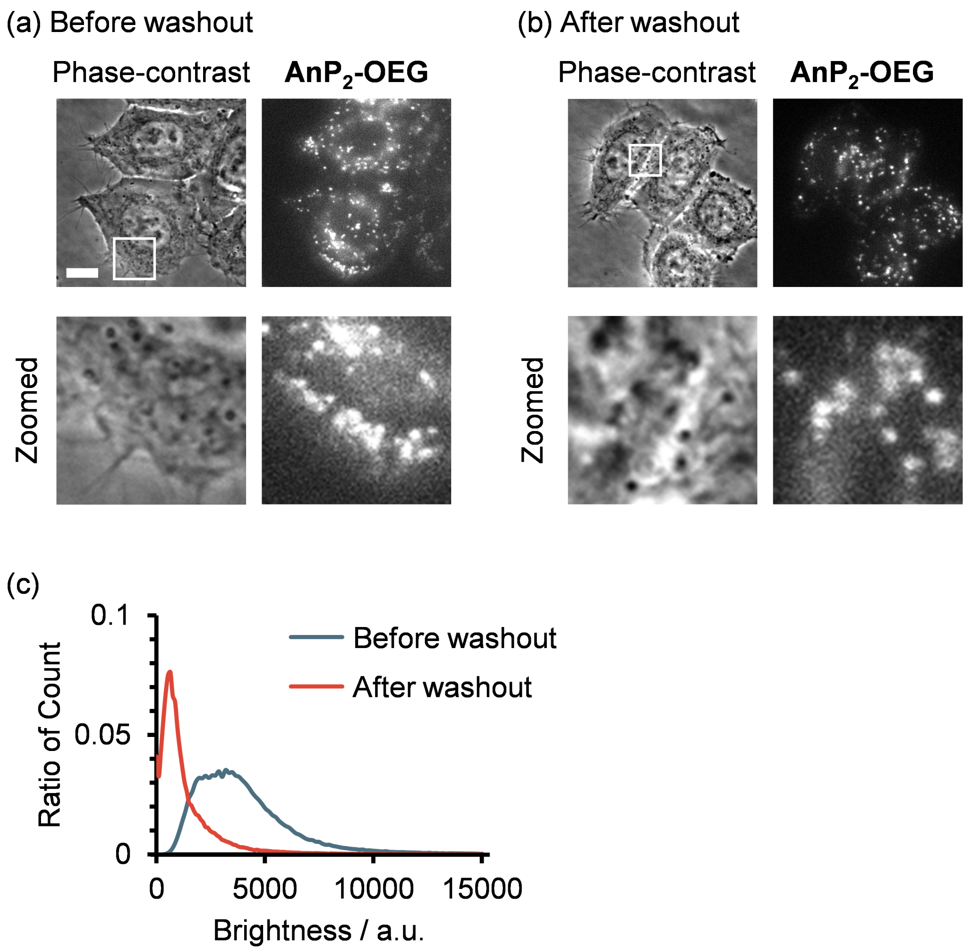

Figure S13. (a) HeLa cells were incubated with 10 μM of **AnP$_2$-OEG** for 80 min (data of "before washout"). (b) Then the cell-culture medium was removed and replaced with **AnP$_2$-OEG**-free medium, followed by incubation for 80 min before microscopic observation (data of "after washout"). Phase-contrast and fluorescence images are shown. Zoomed images of the boxed area are shown on the bottom. Scale bar: 10 μm. (c) Distributions of the brightness in the fluorescent images before and after washout.

7.3 Different staining images of HeLa cells by AnP_2-OEG and MitoTracker

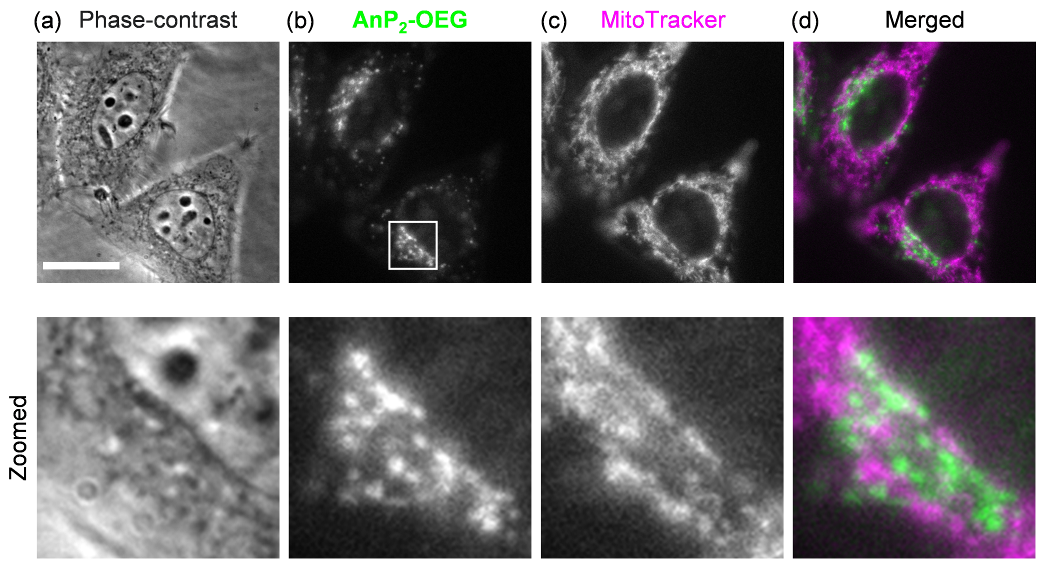

Figure S14. Cells were treated with **AnP_2-OEG** (10 μM) and MitoTracker-Red (50 nM) for 30 min. (a) Phase-contrast images. (b) Fluorescence images from **AnP_2-OEG**. (c) Fluorescence images from MitoTracker-Red. (d) Merged images of (b) and (c). Zoomed images of the boxed area are shown on the bottom. Scale bar: 20 μm.

7.4 Different staining images of HeLa cells by AnP$_2$-OEG and Transferrin-Alexa594

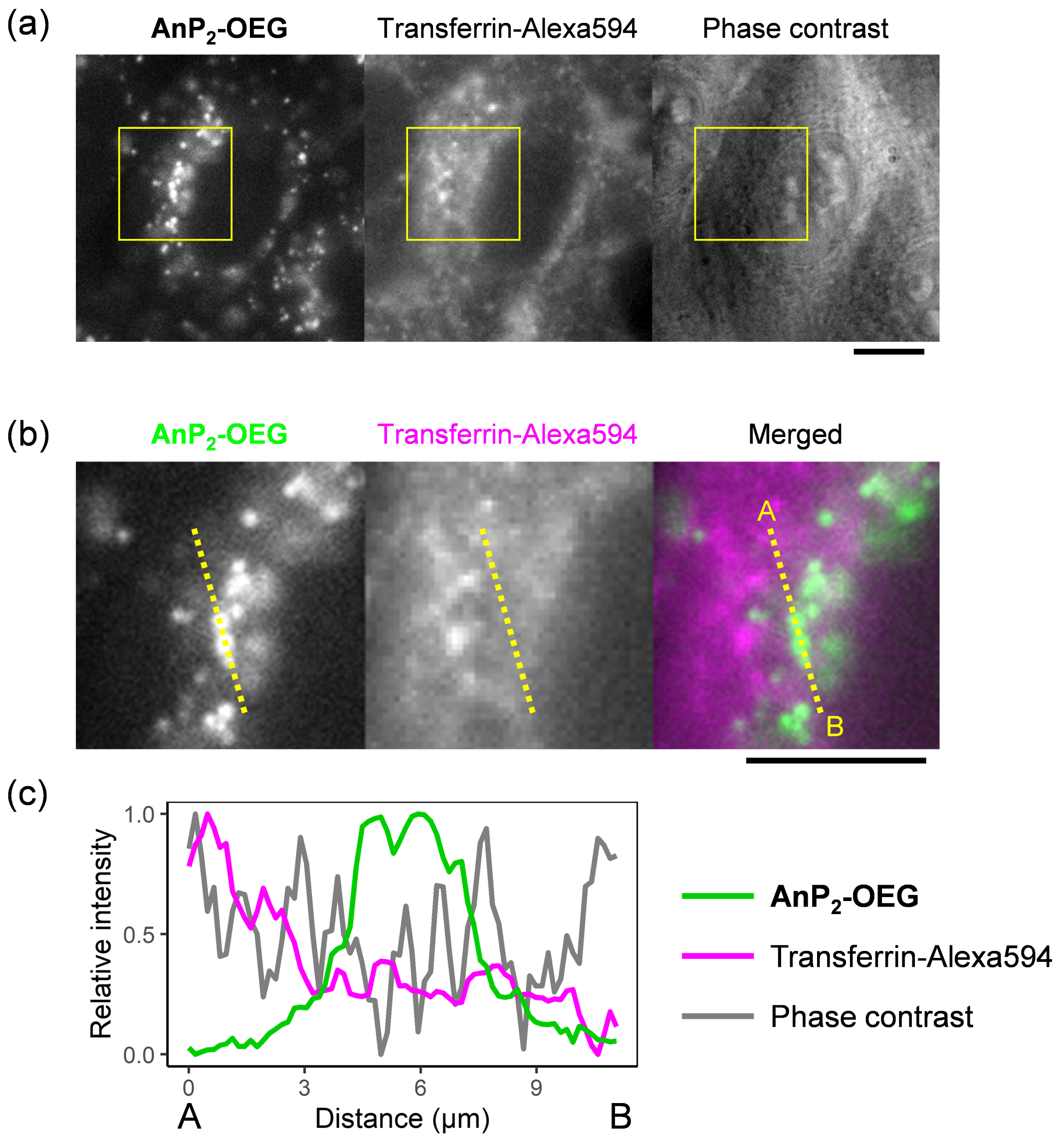

Figure S15. Cells were treated with **AnP$_2$-OEG** (10 μM) and early endosome marker Transferrin-Alexa594 (20 μg/mL) for 15 min. (a) Fluorescence images from **AnP$_2$-OEG** and Transferrin-Alexa594. (b) Zoomed and merged images of the boxed area. (c) Intensity profile along with the dashed lines in (b). Scale bars: 10 μm.

7.5 Different staining images of HeLa cells with aggresomes by AnP_2-OEG and mCherry-ubiquitin

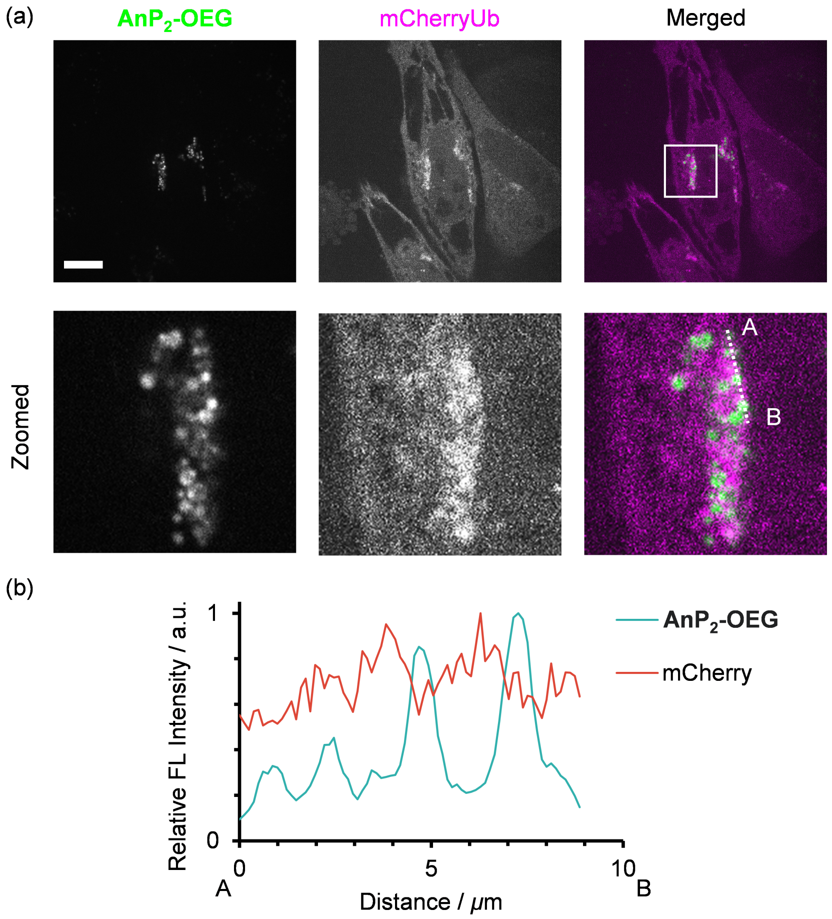

Figure S16. (a) Cells were transfected with plasmids encoding mCherry-ubiquitin and then treated with a proteasome inhibitor MG132 (10 μM) for 6 h to induce aggresome formation. **AnP_2-OEG** (10 μM) was added into the medium for the last 30 min. Images were acquired using an inverted microscope (Ti-E, Nikon) with a PlanApo 100x VC oil immersion objective lens (NA 1.40), a spinning disk unit with a 40 μm pinhole (Dragonfly, Oxford Instruments), a laser unit with 405-nm, 488-nm, and 561-nm laser lines and an EM-CCD camera (iXon Life888, Oxford Instruments). Fluorescence images from **AnP_2-OEG** and mCherry-ubiquitin, merged images of them, and zoomed images of the boxed area are shown on the bottom. Areas with intense mCherry fluorescence indicate aggresomes. Scale bar: 20 μm. (b) Intensity profile of ROIs along the dashed line.

7.6 Co-staining images of B16-F1 melanoma cells by AnP_2-OEG and Tyrp1-mCherry

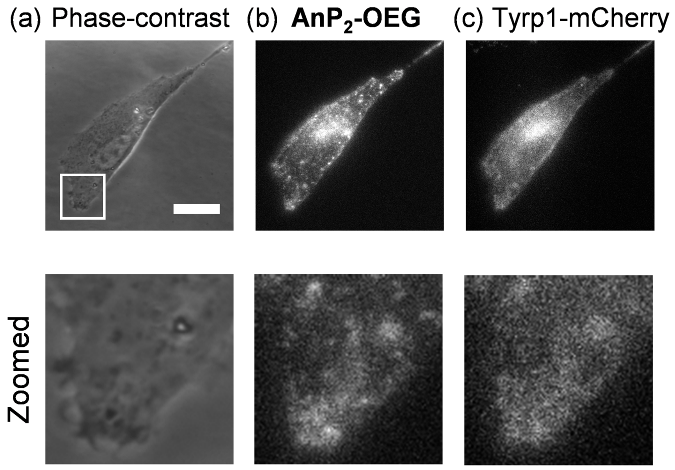

Figure S17. Cells were transfected with plasmids encoding Tyrp1-mCherry, a marker protein for stage III-IV melanosomes, and then treated with **AnP_2-OEG** (10 μM) for 30 min. (a) Phase-contrast images. (b) Fluorescence images from **AnP_2-OEG**. (c) Fluorescence images from Tyrp1-mCherry. The insets are zoomed images of the boxed area. Scale bar: 20 μm.

7.7 Co-localization analysis of AnP_2-OEG with organelle markers

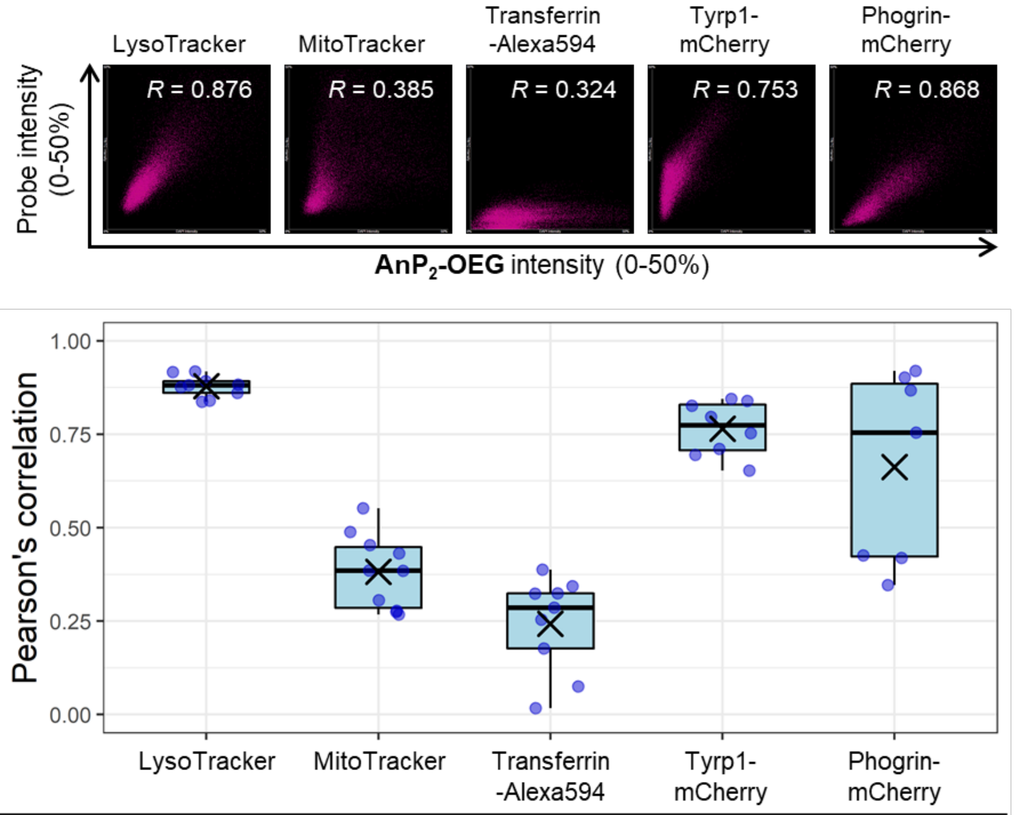

Mean R	0.878	0.382	0.218	0.765	0.662
s. d.	0.029	0.099	0.144	0.073	0.255
Cell number	9	10	10	8	7
Cell line	HeLa	HeLa	HeLa	B16-F1	AtT-20

Figure S18. Pearson's correlation R values of **AnP_2-OEG** and several markers (LysoTracker-Red, MitoTracker™ Red, Transferrin-Alexa594, Tyrp1-mCherry, and Phogrin-mCherry). (Top) Representative correlation plots from single cells. (Middle) Box plots of R values with the mean (×), median (horizontal bar), and individual data points (blue dots). (Bottom) The mean, s. d., the number of analyzed cells, and the cell lines are summarized.

7.8 Effects of osmolality on fluorescence intensity of AnP$_2$-OEG and LysoTracker

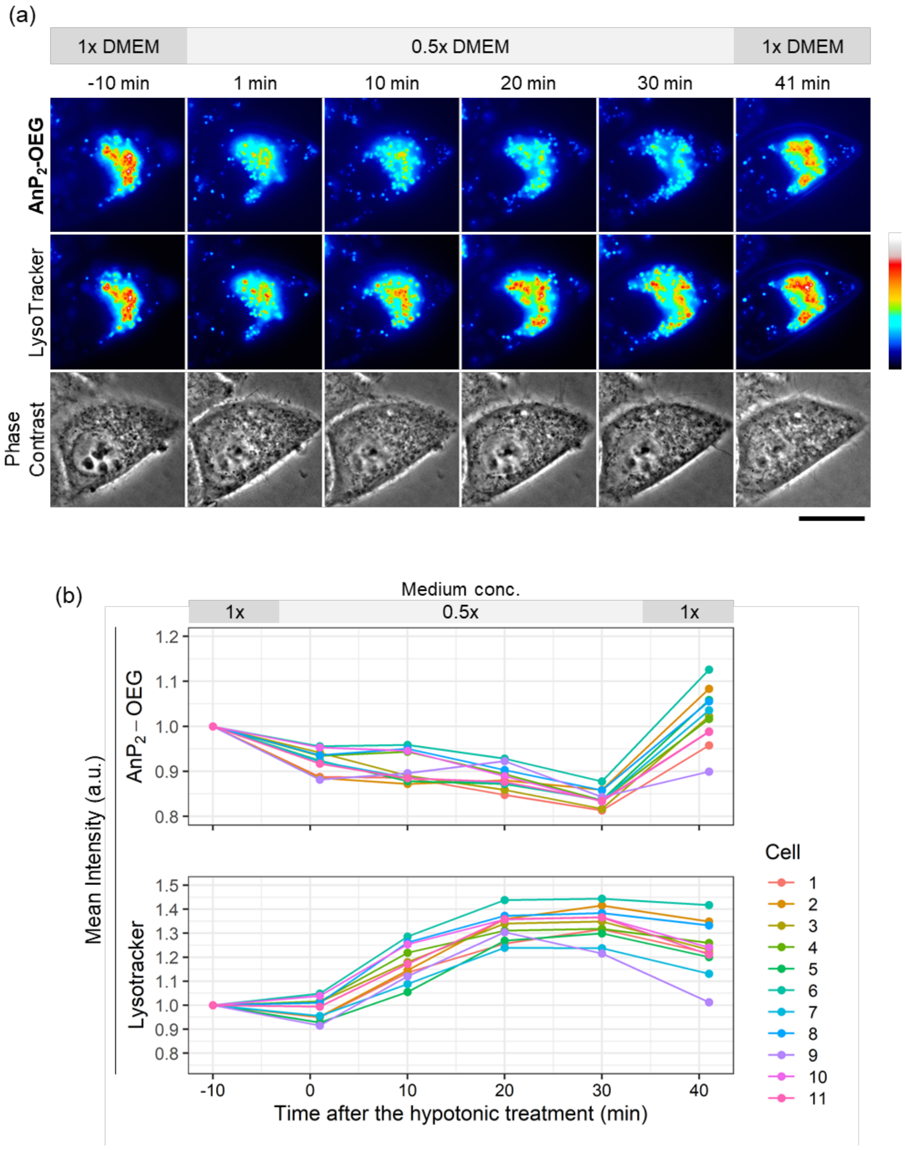

Figure S19. Changes in fluorescence intensity of **AnP$_2$-OEG** and lysotracker in response to changes in osmolality. HeLa cells were stained with 5 μM of **AnP$_2$-OEG** and 50 nM of LysoTracker for 60 min. After fluorescence and phase-contrast images were acquired (-10 min), the culture medium was diluted with an equal volume of H_2O containing **AnP$_2$-OEG** (5 μM) and LysoTracker-Red (50 nM) to incubate cells in a hypotonic condition (0.5× physiological salt concentration) for 40 min, and then the medium was replaced with the normal medium (1× physiological salt concentration). Images were acquired at 1, 10, 20, and 30 min in hypotonic condition and 1 min in the normal medium. (a) Fluorescence and phase-contrast images from **AnP$_2$-OEG** and LysoTracker from -10 to 41 min. Scale bar: 20 μm. Pseudo-color, lookup table (LUT): royal. (b) Relative mean intensity of **AnP$_2$-OEG** and LysoTracker-Red in each cell (N = 11 cells).

7.9 Effects of proteins on fluorescence intensity of AnP$_2$-OEG

We evaluated the dependence of emission intensity on the presence of proteins using the cell lysate and Bovine serum albumin (BSA) (Figure S20), at the concentration range reported for protein-targeting probes (1 μM probe and 10 μM protein).[S10] Here, in the case of BSA, 6 mg mL^{-1} corresponds to 100 μM. In fact, only slight increase in fluorescence enhancement was observed: the intensity remains much weaker than that in 60w% glycerol aqueous solution (*ca.* 11 cP at 293 K,[S5] which is even lower than the reported lysosomal viscosity), suggesting the small influence of proteins on fluorescence enhancement of **AnP$_2$-OEG**.

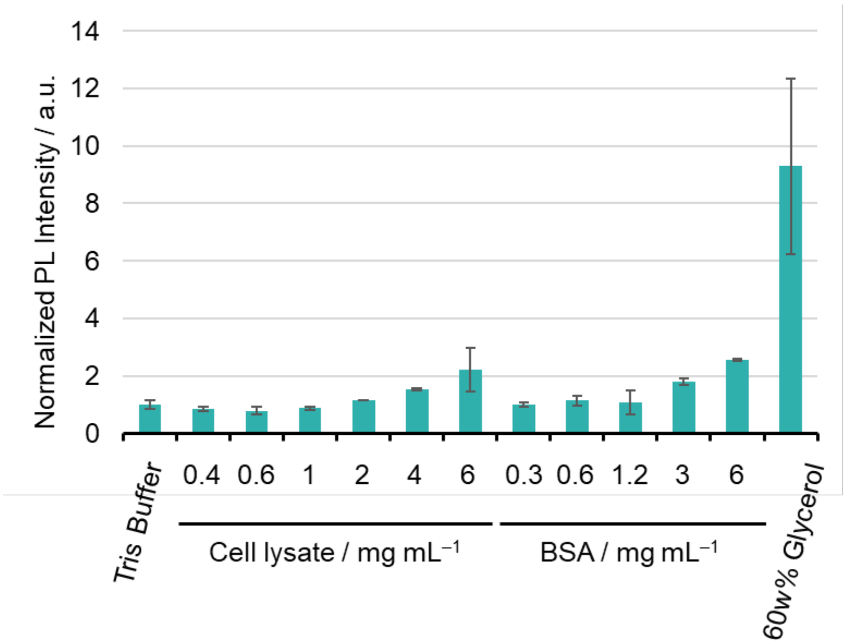

Figure S20. Comparison of emission intensity of **AnP$_2$-OEG** in the presence of proteins (pH 7.4, [**AnP$_2$-OEG**] = 1 μM at room temperature). The error bars indicate standard deviation.

8. References

S1. Wawro, A. M.; Muraoka, T.; Kinbara, K. Chromatography-free synthesis of monodisperse oligo(ethylene glycol) mono-*p*-toluenesulfonates and quantitative analysis of oligomer purity. *Polym. Chem.* **2016**, *7*, 2389–2394.

S2. Haidekker, M. A.; Theodorakis, E. A. Environment-Sensitive Behavior of Fluorescent Molecular Rotors. *J. Biol. Eng.* **2010**, *4*, 11.

S3. Förster, T.; Hoffmann, G. Die Viskositatsabhängigkeit der Fluoreszenzquantenausbeuten einiger Farbstoffsysteme. *Z. Phys. Chem.* **1971**, *75*, 63–76.

S4. Sasaki, S.; Suzuki, S.; Sameera, W. M. C.; Igawa, K.; Morokuma, K.; Konishi, G.-i. Highly Twisted *N*,*N*-Dialkylamines as a Design Strategy to Tune Simple Aromatic Hydrocarbons as Steric Environment-Sensitive Fluorophores. *J. Am. Chem. Soc.* **2016**, *138*, 8194–8206.

S5. Segur, J. B.; Oberstar, H. E. Viscosity of Glycerol and Its Aqueous Solutions. *Int. Eng. Chem.* **1951**, *43*, 2117–2120.

S6. Shirazi, S. G.; Kermanpour, F. Density and Viscosity of 2-Butanol + (1-Propanol, 2-Propanol, or 3-Amino-1-propanol) Mixtures at Temperatures of (293.15 to 323.15) K: Application of the ERAS Model. *J. Chem. Eng. Data* **2019**, *64*, 2292–2302.

S7. Gong, Y.-h.; Shen, C.; Lu, Y.-z.; Meng, H.; Li, C.-x. Viscosity and Density Measurements for Six Binary Mixtures of Water (Methanol or Ethanol) with an Ionic Liquid ([BMIM][DMP] or [EMIM][DMP]) at Atmospheric Pressure in the Temperature Range of (293.15 to 333.15) K. *J. Chem. Eng. Data* **2012**, *57*, 33–39.

S8. Sasaki, S.; Suzuki, S.; Sameera, W. M. C.; Igawa, K.; Morokuma, K.; Konishi, G.-i. Highly Twisted *N*,*N*-Dialkylamines as a Design Strategy to Tune Simple Aromatic Hydrocarbons as Steric Environment-Sensitive Fluorophores. *J. Am. Chem. Soc.* **2016**, *138*, 8194–8206.

S9. Yin, P.-A.; Ou, Q.; Peng, Q.; Shuai, Z. Substituent-controlled aggregate luminescence: Computational unraveling of S_1/S_0 surface crossing. *Aggregate in press*. DOI: 10.1002/agt2.291

S10. For example, see; Zhuang, Y.-D.; Chiang, P.-Y.; Wang, C.-W.; Tan, K.-T. Environment-Sensitive Fluorescent Turn-On Probes Targeting Hydrophobic Ligand-Binding Domains for Selective Protein Detection. *Angew. Chem. Int. Ed.* **2013**, *52*, 8124–8128.
